# *Cadophora* species from marine glaciers in the Qinghai-Tibet Plateau: an example of unsuspected hidden biodiversity

**DOI:** 10.1186/s43008-022-00102-5

**Published:** 2022-09-05

**Authors:** Bingqian Zhang, Xiaoguang Li, Guojie Li, Qi-Ming Wang, Manman Wang

**Affiliations:** 1 Engineering Laboratory of Microbial Breeding and Preservation of Hebei Province, School of Life Sciences, Institute of Life Sciences and Green Development, Hebei University, Baoding, 071002 Hebei China; 2 Science and Technology Division, Hebei University, Baoding, 071002 Hebei China; 3 College of Horticulture, Key Laboratory of Vegetable Germplasm Innovation and Utilization of Hebei, Collaborative Innovation Center of Vegetable Industry in Hebei, Hebei Agricultural University, Baoding, 071001 Hebei China

**Keywords:** Cold-adapted fungi, Dark biodiversity, Marine glacier, Multi-gene phylogeny, *Ploettnerulaceae*

## Abstract

Large numbers of marine glaciers in the Qinghai-Tibet Plateau are especially sensitive to changes of climate and surface conditions. They have suffered fast accumulation and melting and retreated quickly in recent years. In 2017, we surveyed the cold-adapted fungi in these unique habitats and obtained 1208 fungal strains. Based on preliminary analysis of ITS sequences, 41 isolates belonging to the genus *Cadophora* were detected. As one of the most frequently encountered genera, the *Cadophora* isolates were studied in detail. Two phylogenetic trees were constructed: one was based on the partial large subunit nrDNA (LSU) to infer taxonomic placement of our isolates and the other was based on multi-locus sequences of LSU, ITS, TUB and TEF-1*α* to investigate more exact phylogenetic relationships between *Cadophora* and allied genera. Combined with morphological characteristics, nine *Cadophora* species were determined, including seven new to science. Among the new species, only *C. inflata* produces holoblastic conidia and all the others express phialidic conidiogenesis. All isolates have optimum growth temperature at 20 °C or 25 °C*.* With more species involved, the currently circumscribed genus became obviously paraphyletic. All members are clustered into two main clades: one clade mainly includes most of the *Cadophora* species which have phialidic conidiogenesis and we refer to as ‘*Cadophora s. str*.’; the remaining *Cadophora* species have multiform conidiogenesis and are clustered in the second clade, with members of other genera in *Ploettnerulaceae* interspersed among the subclades. The results show a high diversity of *Cadophora* from marine glaciers in the Qinghai-Tibet Plateau and most of them are novel species.

## Introduction

The genus *Cadophora* was first established in 1927, with *C. fastigiata* as the type species, to accommodate dematiaceous hyphomycetes producing solitary phialides with distinct hyaline collarettes (Lagerberg et al. 1927). Due to subtle differences in morphology, Conant (1937) transferred eight *Cadophora* species to the genus *Phialophora*. Later, Gams (2000) proposed that *Phialophora* species related to discomycete sexual morphs of *Mollisia* and related genera belonging to *Helotiales* should be accommodated in *Cadophora*. This proposal was supported by subsequent rDNA sequence analysis of LSU (Harrington and McNew 2003).

Currently, the genus is included in the family *Ploettnerulaceae* of *Helotiales* (Johnston et al. 2019; Ekanayaka et al. 2019) and comprises some species with multiform morphological characters deviated from the original morphological generic concept. For example, *C. antarctica* and *C. fascicularis* produce chains of ramoconidia and conidia on holoblastic conidiogenous cells (Crous et al. 2017; Maciá-Vicente et al. 2020); while *C. obovata* has putatively monoblastic conidiogenous cells that may represent a retrogression of enteroblastic phialidic conidiogenesis and *C. fallopiae* is only observed as a cladophialophora-like synasexual morph in culture (Maciá-Vicente et al. 2020; Crous et al. 2020). Besides, *C. lacrimiformis* only found by its sexual morph, is also included in this asexually typified genus (Ekanayaka et al. 2019). Recent studies based on molecular data have shown that *Cadophora* is apparently paraphyletic and species with distinct morphological variations may share ancestors with other related genera (Maciá-Vicente et al. 2020).

Species of *Cadophora* normally possess multiple trophic modes. They are commonly considered as plant pathogens, root associates or wood and soil colonizers with cosmopolitan distribution. A global survey on the dominant soil fungal communities of different biomes has shown that *Cadophora* is one of the most ubiquitous soil fungal taxa with significantly higher number of genes related to stress-tolerance and resource uptake (Egidi et al. 2019). In some cold Arctic and Antarctic sites, *Cadophora* species have been frequently isolated from soils, marine sediments and organisms, fresh water lakes, especially the historic wood huts and some mummified or submerged drift wood (Blanchette et al. 2004, 2010, 2016; Jurgens et al. 2009; Gonçalves et al. 2012; Furbino et al. 2014; Zhang et al. 2017; Nagano et al. 2017; Duran et al. 2019). They are hypothesized to be key organisms capable of initiating nutrient cycles and energy flows from dead organic materials in high latitudes (Blanchette et al. 2016). Meanwhile, the saprotrophic species, mainly *C. malorum*, *C. luteo-olivacea*, and *C. fastigiata* which were frequently isolated from polar regions are also detected as pathogens or endophytes from different living plants worldwide (Di Marco et al. 2004; Gramaje et al. 2011; Navarrete et al. 2011; Travadon et al. 2015). Enzyme tests of some *Cadophora* members have shown that *C. luteo-olivacea* and *C. malorum* are capable of degrading a range of carbon sources and releasing soluble phosphorus so that their trophic modes could vary depending on their nutrient needs from different substrata (Day and Currah 2011; Walsh et al. 2018).

The Qinghai-Tibet Plateau, lying across the center of Asia and having an average elevation of 4000 m, possesses large numbers of glacial groups that constitute the center of Asian Highland Glaciers. Based on hydrothermal conditions and physical properties, glaciers in China can be divided into continental glaciers and marine glaciers. Continental glaciers, which are also known as cold glaciers, develop in the continental climate areas where precipitation amount is limited; marine glaciers, which are also known as temperate glaciers, generally form in marine climate areas with abundant precipitation (Shi et al. 1964, 2000). Controlled by the marine monsoonal climate, nearly 9000 marine glaciers, which cover a total area of 13,200 square kilometers and account for 22.2% of the total glacier area in China, form at southeast margin of the Qinghai-Tibet Plateau. Under the background of global warming, glaciers all over the world are retreating significantly. In the next 100 years, marine glaciers in the Qinghai-Tibet Plateau, with the features of fast accumulation and melting and being more sensitive to the change of climate, will retreat more quickly (Yao et al. 2004; Chen et al. 2005). It is necessary and urgent to investigate fungal diversity and resources in this unique area.

Our first investigation (2009–2011) on cold-adapted fungi in the permafrost and alpine glaciers of Qinghai-Tibet Plateau indicates that the diversity of cold-adapted fungi from marine glaciers is especially high and many of them may represent unknown species (Wang et al. 2015). Another survey was conducted in 2017, focusing on the diversity of cold-adapted fungi from marine glaciers. Based on preliminary analyses of the generated ITS sequences, 41 strains representing nine *Cadopora* species including seven new species are described and phylogenetic relationships intra and among *Cadophora* and related genera are discussed in this study.

## Materials and methods

### Sample collection

**Fig. 1:**
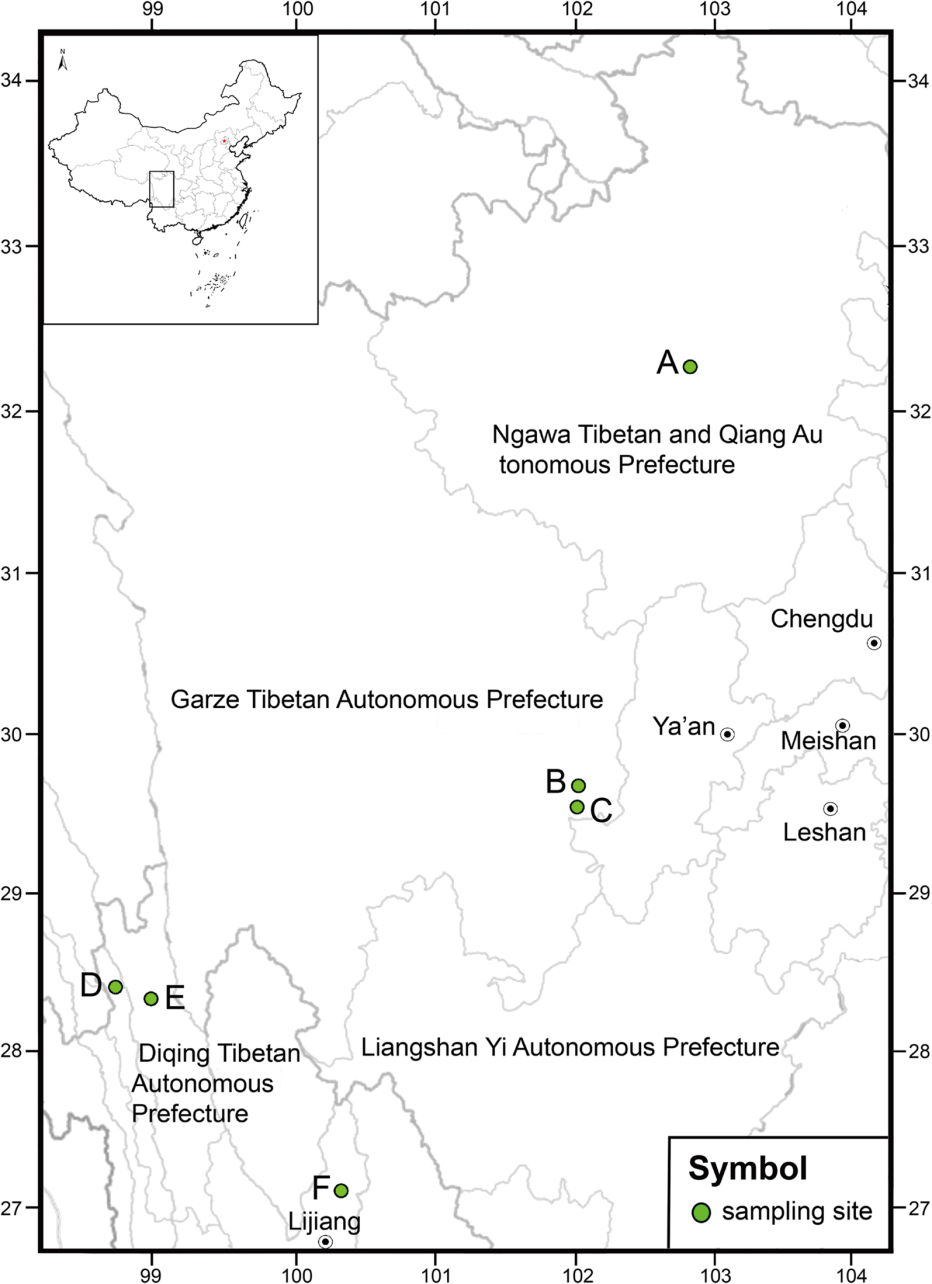
Sampling sites. A. Dagu Glacier; B. Yanzigou Glacier; C. Hailuogou Glacier; D. Mingyong Glacier; E. Baima Snow Mountain; F. Yulong Snow Mountain

**Fig. 2:**
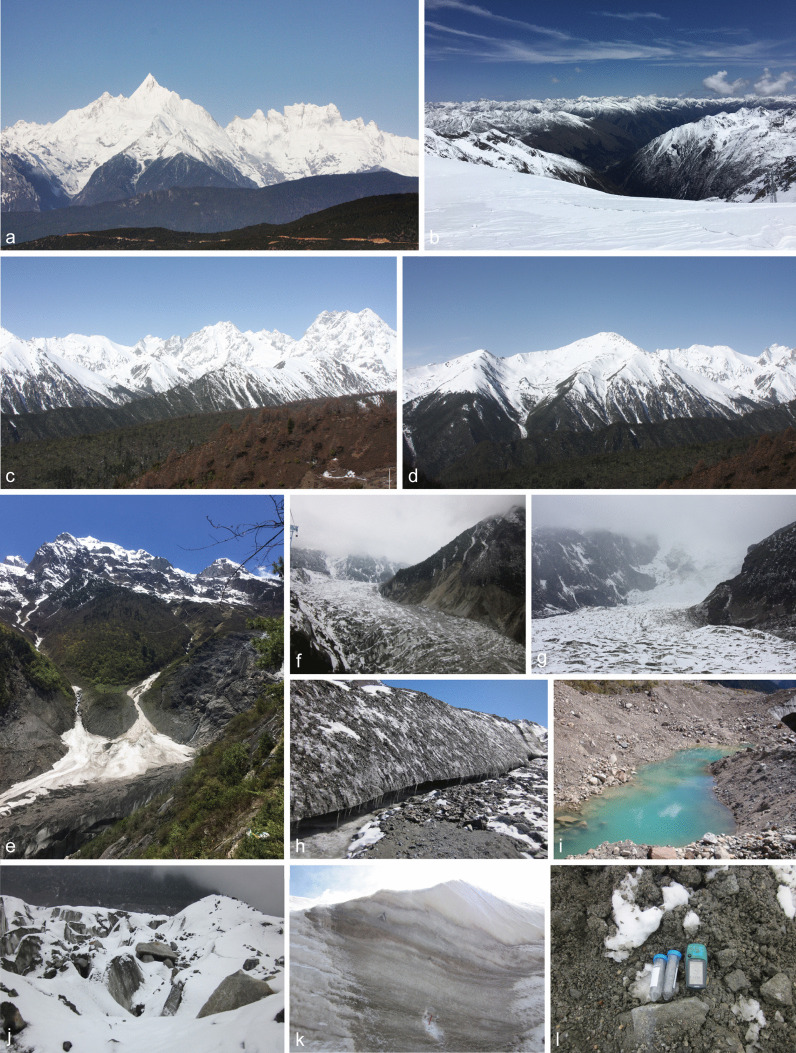
The natural environment of the sampling sites. **a** Meri Snow Mountain (N28°27′25″ E98°45′25″); **b** Dagu Glacier (N32°13′14″ E102°45′29″); **c**, **d** Baima Snow Mountain (N29°23′1″ E99°0′20″); **e** Mingyong Glacier (N28°27′24″ E98°45′51″); **f**–**g** Hailuogou Glacier (N29°33′10″ E101°58′10″); **h**–**l** Details of collecting samples in the glaciers and snow mountains

Soil, ice and water samples were collected from four marine glaciers and two nearby snow-capped mountains in 2017 (Table 1). Sampling sites were selected at different elevations of the following marine glaciers and snow-capped mountains: Hailuogou Glacier, Yanzigou Glacier and Dagu Glacier in Sichuan Province, Yulong Snow Mountain, Baima Snow Mountain and Mingyong Glacier in Yunnan Province (Figs. 1, 2). For all samplings, clean hand tools were surface sterilized with 70% ethanol before use. After the removal of the top 5–10 cm of surface sediment, c. 500 g soil or ice sample was collected from the underlying layer and placed in a fresh Zip-lock plastic bag and sterilized plastic bottles. Melt water samples were directly collected and placed in sterilized centrifuge tubes or Zip-lock plastic bags. All the samples were maintained at 4 °C until arrival at the laboratory.

**Table 1:** Collection details of samples from where *Cadophora* strains were isolated

Sampling location	Collection date	GPS location	Altitude (m)	Substrate
Baima Snow Mountain	10 May 2017	N28°23′29″ E98°59′22″	4125	Soil
		N28°22′59″ E99°0′31″	4343	Soil
		N29°23′1″ E99°0′20″	4366	Soil
Dagu Glacier	1 May 2017	N32°8′19″ E102°56′13″	2380	Soil
		N32°8′19″ E102°56′13″	2380	Water
		N32°15′38″ E102°48′15″	3510	Soil
		N32°14′23″ E102°47′7″	3610	Water
		N32°14′21″ E102°47′5″	3630	Soil
		N32°13′14″ E102°45′29″	4850	Soil
Hailuogou Glacier	28 April 2017	N29°33′10″ E101°58′10″	3180	Water
		N29°34′8″ E101°59′36″	3180	Soil
Mingyong Glacier	9 May 2017	N28°27′25″ E98°45′51″	2960	Water
		N28°27′24″ E98°45′51″	2976	Soil
		N28°27′27″ E98°45′49″	2976	Soil
		N28°27′28″ E98°45′43″	3067	Soil
Yanzigou Glacier	29 April 2017	N29°41′58″ E102°0′7″	2620	Soil
Yulong Snow Mountain	7 May 2017	N27°11′17″ E100°22′43″	3362	Soil
		N27°11′17″ E100°22′43″	3362	Water
		N27°10′52″ E100°19′84″	4531	Soil
		N27°10′55″ E100°19′87″	4531	Soil

### Isolation

Strains were isolated from soil and water samples as soon as they were taken to the lab. Soil samples were isolated with traditional pour plate method: A 10 g quantity of each soil sample was suspended in sterile-distilled water in a flask, the volume was then increased to 100 mL before the suspension was shaken to disperse soil particles and then serially diluted to 10^–2^, 10^–3^ and 10^–4^; 100 mL of each water sample was filtrated by nitrocellulose filter membrane with pore size of 0.45 μm, the membrane with trapped fungi was put in a sterile 50 mL centrifuge tube which contained 10 mL distilled water and the tube was vigorously agitated to suspend the trapped mycelium and spores. About 0.1 mL of each final diluent or concentrate was placed on the surface of two 90 mm diam Petri plates containing 1/4 strength Potato Dextrose Agar (1/4 PDA; 9 g of Potato Dextrose Agar [BD Difco] and 15 g of Agar per L of demineralized water) supplemented with chloramphenicol (0.1 mg/mL) and streptomycin (0.1 mg/mL). The plates were sealed and incubated at 15 °C and 25 °C (one plate per temperature) and were examined for fungal growth at 1 wk intervals for 4 wk. Colonies that appeared on the plates were transferred to two new plates and then incubated at 15 °C and 25 °C. All fungal strains were stored at 4 °C for further studies.

### Morphological studies

41 isolates representing all of the *Cadophora* species isolated were studied in more detail. To enhance sporulation, strains were inoculated on potato dextrose agar (PDA; BD Difco), malt extract agar (MEA, BD Difco) and oatmeal agar (OA; BD Difco). Pine needle medium, H_2_O_2_ treatment and slide culture technique (Xu et al. 2009; Su et al. 2012) were also used to induce sporulation. For phenotypic determination, the strains were transferred to PDA, MEA and OA plates with three replicates and incubated in the dark at 25 °C. Optimal growth temperature (OGT) and maximum growth temperature (MGT) were also tested by culturing each isolate in triplicate on PDA at temperatures ranging from 5 to 35 °C at 5 °C increments. Colony diameters were measured in two perpendicular directions after 2 wk at different temperatures, and the mean diameter was obtained from three replicate plates cultivated at the same temperatures. Colony colors were determined using taxonomic description color charts (Rayner 1970). Microscopic preparations were made by mounting aerial hyphae in water or using the slide cultures directly. Hyphae, conidiophores, and conidia were observed, photographed, and measured with 1000 × magnification by using a Nikon 80i microscope with differential interference contrast (DIC) optics. Specimens were deposited in the Mycological Herbarium of Hebei University (HBU), while living cultures including ex-types were deposited in the China General Microbiological Culture Collection Center (CGMCC).

### DNA extraction, PCR amplification, sequencing and phylogenetic analyses

Genomic DNA was extracted from the fungal mycelia following the protocol described by Wang and Zhuang (2004). The partial large subunit nrDNA (LSU), the internal transcribed spacer region of the nuclear ribosomal RNA gene (ITS), the partial translation elongation factor 1-α gene (TEF-1α) and the β-tubulin (β-TUB) gene were amplified and sequenced with the primer pairs of LROR/LR5 (Vilgalys and Hester 1990), ITS1/ITS4 (White et al. 1990), EF1-688F/EF1-1251R (Alves et al. 2008) and BTCadF/R (Travadon et al. 2015), respectively. PCR was performed in 50 μL reactions containing DNA template 1.0 μL, each forward and reverse primers 1.0 μL, 2 × MasterMix 25 μL (ThermoFisher scientific Co. Ltd., Shanghai, China) and 22 μL H_2_O, PCR parameters were as follows: denaturation at 95 °C for 5 min, followed by 35 cycles of denaturation at 94 °C for 30 s, annealing at a suitable temperature for 30 s, extension at 72 °C for 30 s and a final elongation step at 72 °C for 10 min. Annealing temperature for each gene were 54 °C for LSU and ITS, 51 °C for TEF-1α and 56 °C for TUB. The PCR products were sequenced with the primers mentioned above by BGI Tech Solutions Co., Ltd. (Shenzhen, China).

Nucleotide sequences were initially checked and edited using Chromas software ver. 2.6.6 (http://www.technelysium.com.au/chromas.html) and EdiSeq (Lasergene, DNASTAR) and then were compared to accessions in the GenBank database via BLASTn searching to find the most likely taxonomic designation. To reveal the family placements of the species described in this study, a LSU tree was constructed. To investigate more exact phylogenetic relationships and taxonomic distinctions of novel species, a multi-locus analysis was performed based on ITS, LSU, TUB and TEF1-α genes. Sequence data of the four genes especially those of ex-type strains, were downloaded from GenBank and added to the sequences generated in this study. The datasets were aligned automatically using MAFFT v. 7.471 (Katoh and Standley 2013) and further manual alignment was carried out with MEGA v. 7 (Kumar et al. 2016) and alignments were deposited in TreeBASE (www.treebase.org, submission no. S29383).

Phylogenetic analyses were conducted using Bayesian Inference (BI), Maximum Likelihood (ML) and Maximum Parsimony (MP) methods. For BI analyses, the best fit model of evolution for each partition was estimated by MEGA v. 7. Posterior probabilities were determined by Markov Chain Monte Carlo sampling (MCMC) in MrBayes v. 3.2.7a (Ronquist and Huelsenbeck 2003) using the estimated models of evolution. For the LSU/multi-locus trees, six simultaneous Markov chains were run for 4,000,000/8,000,000 generations and trees were sampled every 100th generation (resulting in 40,000/80,000 total trees). The first 10,000/20,000 trees represented the burn-in phase of the analyses were discarded and the remaining 30,000/60,000 trees were used for posterior probabilities (PP) calculation in the majority rule consensus trees. The ML analyses were performed by raxmlGUI 2.0.0-beta (Edler et al. 2019) using the GTRGAMMA model with the rapid bootstrapping and search for best scoring ML tree algorithm, including 1000 bootstrap replicates. The MP analyses were conducted using PAUP v. 4.0b10 (Swofford 2002) and an unweighted parsimony (UP) analysis was performed. Trees were inferred using the heuristic search option with TBR branch swapping and 1000 random sequence additions. Branches of zero length were collapsed and all equally most parsimonious trees were saved. Descriptive tree statistics such as tree length (TL), consistency index (CI), retention index (RI), rescaled consistency index (RC) and homoplasy index (HI), were calculated for trees generated. Clade stability was assessed using bootstrap analysis with 1000 replicates, each with 10 replicates of random stepwise addition of taxa.

## Results

1208 fungal strains isolated from 120 samples of four glaciers and two snow-capped mountains were preliminarily identified based on BLAST comparison of ITS sequences against the GenBank database. As one of the most commonly encountered fungal groups, 41 isolates belonging to *Cadophora* were studied in detail.

### Phylogenetic analyses

Sequences of referential species, especially those of ex-type strains, were retrieved from GenBank and added to the sequences generated in this study (Table 2). The alignments of partial sequences of LSU (For LSU phylogenetic analysis), ITS, LSU (For muti-locus phylogenetic analysis), TUB and TEF1-α have 855, 452, 834, 582 and 694 characters, respectively.

**Table 2:** Strains analyzed in this study, with collection details and GenBank accession numbers

Species	Strain no	Host/substrate	Country	GenBank Accession No			
				LSU	ITS	TUB	TEF1-α
*Articulospora tetracladia*	DSM 104345	–	–	MK226456	MH930816	MK241460	MK241447
*Ascocorticium anomalum*	CBS 874.71	–	Germany	MH872135	–	–	–
*Cadophora africana*	CBS 120890^T^	*Prunus salicina*, necrotic wood	South Africa	MT156170	MN232936	MN232967	MN232988
*Cadophora antarctica*	FMR16056^T^	Diesel-contaminated soil sample	Antarctica	MG385663	MG385664	–	–
*Cadophora bubakii*	CBS 198.30^T^	Margarine	Czech Republic	MH866559	MH855111	–	MN232989
***Cadophora caespitosa***	**CGMCC3.20179 = MY156**^**T**^	**Water in Mingyong Glacier**	**China**	**MT908194**	**MT889936**	**MT921201**	**MT900568**
***Cadophora caespitosa***	**CGMCC3.20180 = MY169**	**Water in Mingyong Glacier**	**China**	**MT908195**	**MT889937**	**MT921202**	**MT921172**
***Cadophora caespitosa***	**CGMCC3.20192 = DG1120**	**Water in Dagu Glacier**	**China**	**MT908222**	**MT889964**	**MT921229**	**MT921197**
***Cadophora caespitosa***	**CGMCC3.20431 = HL674**	**Water in Hailuogou Glacier**	**China**	**MW793546**	**MW793520**	**MW818434**	**MW810619**
***Cadophora caespitosa***	**CGMCC3.20432 = BM691**	**Soil in Baima Snow Mountain**	**China**	**MW793547**	**MW793521**	**MW818435**	**MW810620**
*Cadophora constrictospora*	P1751^T^	Endophytic in roots of *Microthlaspi*	Bulgaria	MN339369	KT269023	–	MN325874
***Cadophora daguensis***	**CGMCC3.20845 = DG5**	**Soil in Dagu Glacier**	**China**	**OL477357**	**OL477351**	**OL674144**	**OL674147**
***Cadophora daguensis***	**CGMCC3.20846 = DG21**^**T**^	**Soil in Dagu Glacier**	**China**	**OL477356**	**OL714365**	**OL674143**	**OL674146**
*Cadophora dextrinospora*	AG5	Decayed wood in *Anoplophora glabripennis* galleries	Finland	–	MF188986	–	–
*Cadophora dextrinospora*	CBS 401.78^T^	Decaying wood	Spain	MH872917	NR_119489	–	–
*Cadophora domestica*	CBS 146265^T^	From necrotic tissues from crown of *Prunus domestica* (*Rosaceae*) nursery tree	South Africa	–	MN873024	MN873028	MN873031
*Cadophora fallopiae*	CPC 35,742	*Reynoutria japonica*	Germany	MT223877	MT223782	–	–
*Cadophora fascicularis*	P2794^T^	Endophytic in roots of *Microthlaspi erraticum*	Germany	MN339414	KT269992	–	MN325918
*Cadophora fastigiata*	CBS 307.49	Pine wood	Sweden	MH868062	MH856538	KM497131	KM497087
*Cadophora ferruginea*	P1323^T^	Endophytic in roots of *Microthlaspi perfoliatum*	Spain	MN339356	KT268618	–	MN325861
*Cadophora gregata*	ATCC 11073^T^	*Glycine max*, brown stem rot	Japan	MF979571	U66731	MF677920	MF979586
*Cadophora helianthi*	CBS 144752^T^	*Helianthus annuus*, necrotic tissue in stem	Ukraine	–	MK813837	MH733391	MH719029
***Cadophora indistincta***	**CGMCC3.20233 = DG978**	**Soil in Dagu Glacier**	**China**	**MT908210**	**MT889952**	**MT921217**	**MT921186**
***Cadophora indistincta***	**CGMCC3.20234 = DG1054**	**Water in Dagu Glacier**	**China**	**MT908215**	**MT889957**	**MT921222**	**MT921191**
***Cadophora indistincta***	**CGMCC3.20189 = DG1014**^**T**^	**Water in Dagu Glacier**	**China**	**MT908211**	**MT889953**	**MT921218**	**MT921187**
***Cadophora indistincta***	**CGMCC3.20195 = DG1017**	**Soil in Dagu Glacier**	**China**	**MT908212**	**MT889954**	**MT921219**	**MT921188**
***Cadophora indistincta***	**CGMCC3.20196 = DG1074**	**Soil in Dagu Glacier**	**China**	**MT908219**	**MT889961**	**MT921226**	**MT921194**
***Cadophora inflata***	**CGMCC3.20186 = MY759**^**T**^	**Soil in Mingyong Glacier**	**China**	**MT908204**	**MT889946**	**MT921211**	**MT921181**
*Cadophora interclivum*	CBS143323 = BAG4^T^	*Carex sprengelii*, root	Canada	MF979565	MF979577	MF677917	MF979583
*Cadophora lacrimiformis*	MFLU 16-1486^T^	Unknown *Brassicaceae*, dead stem	Russia	MK591959	MK585003	–	–
*Cadophora luteo-olivacea*	CBS 141.41^T^	Waste water	Sweden	MH867586	MH856092	KM497133	JN808856
*Cadophora luteo-olivacea*	GLMC 517	*Prunus domestica*, necrotic wood	Germany	–	MN232937	MN232968	MN233003
***Cadophora magna***	**CGMCC3.20188 = MY902**^**T**^	**Soil in Mingyong Glacier**	**China**	**MT908208**	**MT889950**	**MT921215**	**MT921184**
*Cadophora malorum*	CBS 165.42	*Amblystoma mexicanum*	Netherlands	MH867607	MH856109	KM497134	KM497090
***Cadophora malorum***	**CGMCC3.20184 = YL412**	**Soil in Yulong Snow Mountain**	**China**	**MT908200**	**MT889942**	**MT921207**	**MT921177**
*Cadophora margaritata*	CBS 144,084	Colonized wood	Finland	–	MH203866	–	–
*Cadophora margaritata*	CBS144083^T^	Colonized wood	Finland	MH267288	KJ702027	MH327786	–
*Cadophora melinii*	CBS 268.33^T^	Probably wood-pulp	Sweden	MH866887	NR_111150	KM497132	KM497088
*Cadophora melinii*	ONC1	*Vitis vinifera* 'Cabernet Franc', wood canker	Canada	–	KM497033	KM497114	KM497070
*Cadophora melinii*	U11	*Vitis vinifera* 'Sangiovese', vascular discoloration	USA	–	KM497032	KM497113	KM497069
*Cadophora meredithiae*	CBS143322 = BAG2^T^	*Carex sprengelii,* root	Canada	MF979568	MF979574	MF677914	MF979580
*Cadophora neoregeliae*	CBS 146821^T^	From leaf spots of *Neoregelia* sp.	New Zealand	MZ064468	MZ064411	–	–
*Cadophora novi-eboraci*	GLMC 239	*Prunus cerasus*, necrotic wood	Germany	–	MN232942	MN232973	MN232990
*Cadophora novi-eboraci*	GLMC 273	*Prunus cerasus*, necrotic wood	Germany	MT156177	MN232943	MN232974	MN232991
*Cadophora novi-eboraci*	NYC14^T^	*Vitis labruscana*, wood canker	USA	–	KM497037	KM497118	KM497074
***Cadophora novi-eboraci***	**CGMCC3.20190 = YZ1034**	**Soil in Yanzigou Glacier**	**China**	**MT908213**	**MT889955**	**MT921220**	**MT921189**
***Cadophora novi-eboraci***	**CGMCC3.20434 = YZ1026**	**Soil in Yanzigou Glacier**	**China**	**MW793552**	**MW793526**	**MW818436**	**MW810622**
*Cadophora obovata*	P1963^T^	Endophytic in roots of *Microthlaspi erraticum*	Germany	MN339384	KT269230	–	MN325888
*Cadophora orientoamericana*	CTC5	*Vitis* hybrid 'Cayuga white', wood canker	USA	–	KM497015	KM497096	KM497052
*Cadophora orientoamericana*	MYA-4972 = NHC1^T^	*Vitis vinifera* ‘Niagara’	USA	MF979573	KM497018	KM497099	KM497055
*Cadophora prunicola*	CBS 120891^T^	*Prunus salicina*, necrotic wood	South Africa	MT156182	MN232949	MN232979	MN232997
*Cadophora prunicola*	GLMC 276	*Prunus cerasus*, necrotic wood	Germany	–	MN232951	MN232980	MN232998
***Cadophora qinghai-tibetana***	**CGMCC3.20181 = BM327**	**Soil in Baima Snow Mountain**	**China**	**MT908197**	**MT889939**	**MT921204**	**MT921174**
***Cadophora qinghai-tibetana***	**CGMCC3.20182 = YL357**	**Water in Yulong Snow Mountain**	**China**	**MT908198**	**MT889940**	**MT921205**	**MT921175**
***Cadophora qinghai-tibetana***	**CGMCC3.20183 = BM360**	**Soil in Baima Snow Mountain**	**China**	**MT908199**	**MT889941**	**MT921206**	**MT921176**
***Cadophora qinghai-tibetana***	**CGMCC3.20185 = MY474**	**Soil in Mingyong Glacier**	**China**	**MT908202**	**MT889944**	**MT921209**	**MT921179**
***Cadophora qinghai-tibetana***	**CGMCC3.20191 = DG1048**	**Soil in Dagu Glacier**	**China**	**MT908214**	**MT889956**	**MT921221**	**MT921190**
***Cadophora qinghai-tibetana***	**CGMCC3.20193 = DG1156**^**T**^	**Soil in Dagu Glacier**	**China**	**MT908223**	**MT889965**	**MT921230**	**MT921198**
***Cadophora qinghai-tibetana***	**CGMCC3.20194 = YL414**	**Water in Yulong Snow Mountain**	**China**	**MT908201**	**MT889943**	**MT921208**	**MT921178**
***Cadophora qinghai-tibetana***	**CGMCC3.20197 = DG1105**	**Soil in Dagu Glacier**	**China**	**MT908221**	**MT889963**	**MT921228**	**MT921196**
***Cadophora qinghai-tibetana***	**CGMCC3.20228 = YL73**	**Soil in Yulong Snow Mountain**	**China**	**MT908193**	**MT889905**	**MT921200**	**MT898424**
***Cadophora qinghai-tibetana***	**CGMCC3.20229 = YL319**	**Water in Yulong Snow Mountain**	**China**	**MT908196**	**MT889938**	**MT921203**	**MT921173**
***Cadophora qinghai-tibetana***	**CGMCC3.20230 = BM523**	**Soil in Baima Snow Mountain**	**China**	**MT908203**	**MT889945**	**MT921210**	**MT921180**
***Cadophora qinghai-tibetana***	**CGMCC3.20231 = MY873**	**Soil in Mingyong Glacier**	**China**	**MT908207**	**MT889949**	**MT921214**	**MT921183**
***Cadophora qinghai-tibetana***	**CGMCC3.20232 = DG975**	**Soil in Dagu Glacier**	**China**	**MT908209**	**MT889951**	**MT921216**	**MT921185**
***Cadophora qinghai-tibetana***	**CGMCC3.20235 = DG1073**	**Soil in Dagu Glacier**	**China**	**MT908218**	**MT889960**	**MT921225**	**MT921193**
***Cadophora qinghai-tibetana***	**CGMCC3.20236 = DG1087**	**Soil in Dagu Glacier**	**China**	**MT908220**	**MT889962**	**MT921227**	**MT921195**
***Cadophora qinghai-tibetana***	**CGMCC3.20433 = BM857**	**Soil in Baima Snow Mountain**	**China**	**MW793551**	**MW793525**	**MW818439**	**MW810621**
***Cadophora qinghai-tibetana***	**CGMCC3.20435 = YL305**	**Water in Yulong Snow Mountain**	**China**	**MW793548**	**MW793522**	**MW818433**	**–**
***Cadophora qinghai-tibetana***	**CGMCC3.20436 = BM816**	**Soil in Baima Snow Mountain**	**China**	**MW793550**	**MW793524**	**MW818438**	**–**
***Cadophora qinghai-tibetana***	**CGMCC3.20437 = HL876**	**Soil in Hailuogou Glacier**	**China**	**MW793549**	**MW793523**	**MW818437**	**–**
***Cadophora qinghai-tibetana***	**CGMCC3.20847 = MY492**	**Soil in Mingyong Glacier**	**China**	**OL477358**	**OL477352**	**OL674145**	**OL674148**
***Cadophora qinghai-tibetana***	**CGMCC3.20848 = MY527**	**Soil in Mingyong Glacier**	**China**	**OL815016**	**OL815013**	**OL790381**	**OL790384**
***Cadophora qinghai-tibetana***	**CGMCC3.20849 = MY588**	**Soil in Mingyong Glacier**	**China**	**OL815017**	**OL815014**	**OL790382**	**OL790385**
***Cadophora qinghai-tibetana***	**CGMCC3.20850 = MY589**	**Soil in Mingyong Glacier**	**China**	**OL815018**	**OL815015**	**OL790383**	**OL790386**
*Cadophora ramosa*	CBS 111,743	*Actinidia chinensis*, vascular discoloration	Italy	–	DQ404351	KM497136	KM497091
*Cadophora ramosa*	GLMC 377^T^	*Prunus cerasus*, necrotic wood	Germany	MT156187	MN232956	MN232984	MN233002
*Cadophora rotunda*	CBS 146264^T^	From necrotic tissues from crown of *Prunus domestica* (*Rosaceae*) nursery tree	South Africa	–	MN873023	MN873029	MN873030
*Cadophora sabaouae*	WAMC117	*Vitis vinifera*	Algeria	–	MT524745	MT646750	MT646747
*Cadophora sabaouae*	WAMC118	*Vitis vinifera*	Algeria	–	MT524744	MT646751	MT646748
*Cadophora sabaouae*	WAMC34^T^	*Vitis vinifera*	Algeria	–	MT644187	MT646749	MT646746
*Cadophora vinacea*	CBS 146263^T^	From necrotic tissue in trunk of *Vitis vinifera* cv. Ehrenfelser (*Vitaceae*)	Canada	–	MN873025	MN873027	MN873032
*Cadophora viticola*	Cme-1	*Vitis vinifera* 'Syrah', black streaks in shoots	Spain	–	HQ661096	HQ661096	HQ661081
*Cadophora viticola*	Cme-2^T^	*Vitis vinifera* 'Syrah', black streaks in shoots	Spain	–	HQ661097	HQ661097	HQ661082
*Cadophora vivarii*	CBS 146262^T^	From necrotic tissue of bud union of *Malus domestica* (*Rosaceae*) nursery tree	South Africa	–	KY312633	MN873026	MN873033
***Cadophora yulongensis***	**CGMCC3.20187 = YL814**^**T**^	**Soil sample in Yulong Snow Mountain**	**China**	**MT908206**	**MT889948**	**MT921213**	**MT921182**
*Calycina alstrupii*	Pz162^T^	On *Lobaria pulmonaria* growing on trunk of *Alnus incana*	Norway	KY305097	–	–	–
*Calycina marina*	TROM F26093	Dead seaweed (*Ascophyllum nodosum*)	Norway	KT185670	–	–	–
*Cenangium acuum*	TAAM 198,449	*Pinus sylvestris*	Czech Republic	KX090828	–	–	–
*Cenangium ferruginosum*	CBS 556.70	–	Netherlands	MH871625	–	–	–
*Chaetomella acutiseta*	AFTOL-ID 270	–	–	AY544679	–	–	–
*Chaetomella oblonga*	CBS 110.78	Leaf of *Acer* sp.	Canada	MH872875	–	–	–
*Chlorociboria aeruginosa*	CBS 139.28	–	–	MH877688	–	–	–
*Chlorociboria clavula*	D1611	–	New Zealand	JN939941	–	–	–
*Collembolidpora disimillis*	CBS 146372^T^	*Microthlaspi erraticum*	Bulgaria	MN339373	KT269125	–	MN325878
*Collembolidpora disimillis*	P1924	*Microthlaspi erraticum*	Germany	MN339378	KT269192	–	MN325882
*Collembolispora aristata*	CPC21145^T^	Foam in an unnamed right tributary of the brook Bezenek	Czech Republic	KC005811	NR_111830	–	KC005818
*Collembolispora barbata*	CBS 115,944 = UMB-088.01^T^	Mountain freshwater stream	Portugal	–	NR_111443	–	–
*Cordierites frondosa*	HKAS41508	–	–	AY789354	–	–	–
*Cordierites guianensis*	192	–	–	EU107270	–	–	–
*Cudoniella clavus*	AFTOL-ID 166	–	–	DQ470944	–	–	–
*Dermea bicolor*	CBS 135.46	–	Canada	MH867659	–	–	–
*Dermea cerasi*	CBS 432.67	–	–	MH870721	–	–	–
*Graphium rubrum*	CBS 210.34^T^	–	USA	MH866974	–	–	–
*Helgardia anguioides*	CBS 496.80^T^	–	Germany	MH873055	–	–	–
*Helgardia anguioides*	RAN45	–	Germany	–	AY266144	–	–
*Hyaloscypha finlandica*	CBS 444.86^T^	*Pinus sylvestris*, root of seedling	Finland	MH873675	NR_121279	KM497130	KM497086
*Hyaloscypha melinii*	CBS 143705^T^	–	Czech Republic	NG_068558	–	–	–
*Hyaloscypha vitreola*	CBS 126,276	–	Finland	MH875413	–	–	–
*Hymenula cerealis*	CBS 132.34^T^	*Triticum aestivum*, culm	Japan	NG_070839	NR_171209	–	–
*Lachnum carneolum*	CBS 231.54	–	France	MH868838	–	–	–
*Lachnum diminutum*	CBS 232.54	–	France	MH868839	–	–	–
*Leotia lubrica*	KKM 427	Mycorrhizal root tip	Costa Rica	KF836631	–	–	–
*Leptodophora echinata*	P1518	*Microthlaspi erraticum*	Croatia	MN339364	KT268812	–	MN325869
*Leptodophora echinata*	P6045^T^	Endophytic in roots of *Microthlaspi perfoliatum*	Spain	MN339428	KT270239	–	MN325932
*Leptodophora gamsii*	P2440	*Microthlaspi erraticum*	France	MN339395	KT269671	–	MN325900
*Leptodophora gamsii*	P2437^T^	Endophytic in roots of *Microthlaspi erraticum*	France	–	KT269668	–	MN325899
*Leptodophora orchidicola*	CBS 146,385	*Arabidopsis thaliana*	Netherlands	MN365743	MN365799	–	MN325937
*Leptodophora orchidicola*	UAMH 8152	*Pedicularis bracteosa*, root	Canada	MF979572	AF214576	MF677921	MF979587
*Leptodophora variabilis*	P1176^T^	Endophytic in roots of *Microthlaspi perfoliatum*	Croatia	MK539845	KT268493	–	MK550890
*Leptodophora variabilis*	P1331	*Microthlaspi perfoliatum*	Spain	MK539836	KT268626	–	MK550891
*Mastigosporium album*	CPC 22945^T^	*Alopecurus pratensis*	Netherlands	KJ710451	KJ710476	–	–
*Mastigosporium kitzebergense*	CBS 270.69^T^	–	Germany	MH871040	MH859306	–	–
*Mollisia cinerea*	CBS 122,029	Fallen log	USA	MT026558	–	–	–
*Mollisia cinerella*	CBS 312.61	–	France	MH869631	MH858062	–	–
*Mollisia discolor*	CBS 289.59	–	France	MT026504	–	–	–
*Mollisia fallens*	CBS 221.56	–	Netherlands	MT026505	–	–	–
*Mycochaetophora gentianae*	MAFF 239231^T^	–	Japan	AB496937	NR_121201	–	–
*Mycochaetophora* sp.	MAFF 239,284	–	Japan	AB469680	AB469681	–	–
*Neospermospora avenae*	CBS 227.38^T^	*Avena sativa*	USA	NG_077377	MW298276	–	–
*Oculimacula acuformis*	CBS 495.80^T^	Culm base	Germany	MH873054	MH861289	–	MG934497
*Oculimacula aestiva*	CBS 114,730	–	Sweden	–	MG934454	–	MG934496
*Oculimacula yallundae*	CBS 128.31	–	France	–	MH855154	–	MG934499
*Oculimacula yallundae*	CBS 494.80	Culm base	Germany	–	JF412009	–	MG934500
*Phialocephala dimorphospora*	CBS 976.72	–	Germany	MH878299	–	–	–
*Phialophora dancoi*	CBS 329.90^T^	–	Argentina	MH873899	MH862214	–	–
*Pleuroascus nicholsonii*	CBS 345.73^T^	The dung of pack rat	USA	MH872404	–	–	–
*Porodiplodia livistonae*	CPC 32154^T^	*Livistona australis*	Australia	NG_069575	–	–	–
*Porodiplodia vitis*	CBS 144634^T^	*Vitis vinifera*	USA	MK442552	–	–	–
*Rhexocercosporidium camporesii*	MFLU 17-1594^T^	Dead stems	Italy	MN688632	MN688634	–	–
*Rhexocercosporidium carotae*	CBS 418.65^T^	–	Norway	MH870289	NR_111086	–	–
*Rhexocercosporidium microsporum*	MFLU 18-2672^T^	Unknown *Apiaceae*, stem	UK	MK591966	MK584939	–	–
*Rhynchosporium agropyri*	H11	–	–	–	HM627478	–	HM627463
*Rhynchosporium commune*	H10	–	–	–	–	HM627437	HM627462
*Rhynchosporium commune*	H7	–	–	–	–	HM627434	HM627459
*Rhynchosporium orthosporum*	04CH-Bar-A.1.1.3	Dactylis glomerata	Switzerland	KU844335	–	–	–
*Rhynchosporium secalis*	02CH4-6a.1	–	Switzerland	–	KU844333	–	–
*Rutstroemia bulgarioides*	TAAM 198,322	Fallen cone	Estonia	KX090836	–	–	–
*Rutstroemia firma*	CBS 115.86^T^	–	Netherlands	MH873619	–	–	–
*Sclerotinia bulborum*	CBS 297.31	–	USA	MH866668	–	–	–
*Sclerotinia sclerotiorum*	WZ0067	–	China	AY789347	–	–	–
*Xylaria hypoxylon*	CBS 120.16	–	–	MH866173	–	–	–
*Ypsilina buttingtonensis*	CPC 39109^T^	From heartwood of 1000-yr-old *Quercus* sp.	UK	MT373355	MT373372	–	–
*Ypsilina graminea*	CBS 114630^T^	–	UK	MH874529	NR_160217	–	–

^T^ex-type strain; ^1^LSU: large subunit nrDNA; ITS: Internal transcribed spacers 1 and 2 together with 5.8S nrDNA; TUB: partial beta-tubulin gene; TEF1-α: partial translation elongation factor 1-alpha gene

**Fig. 3:**
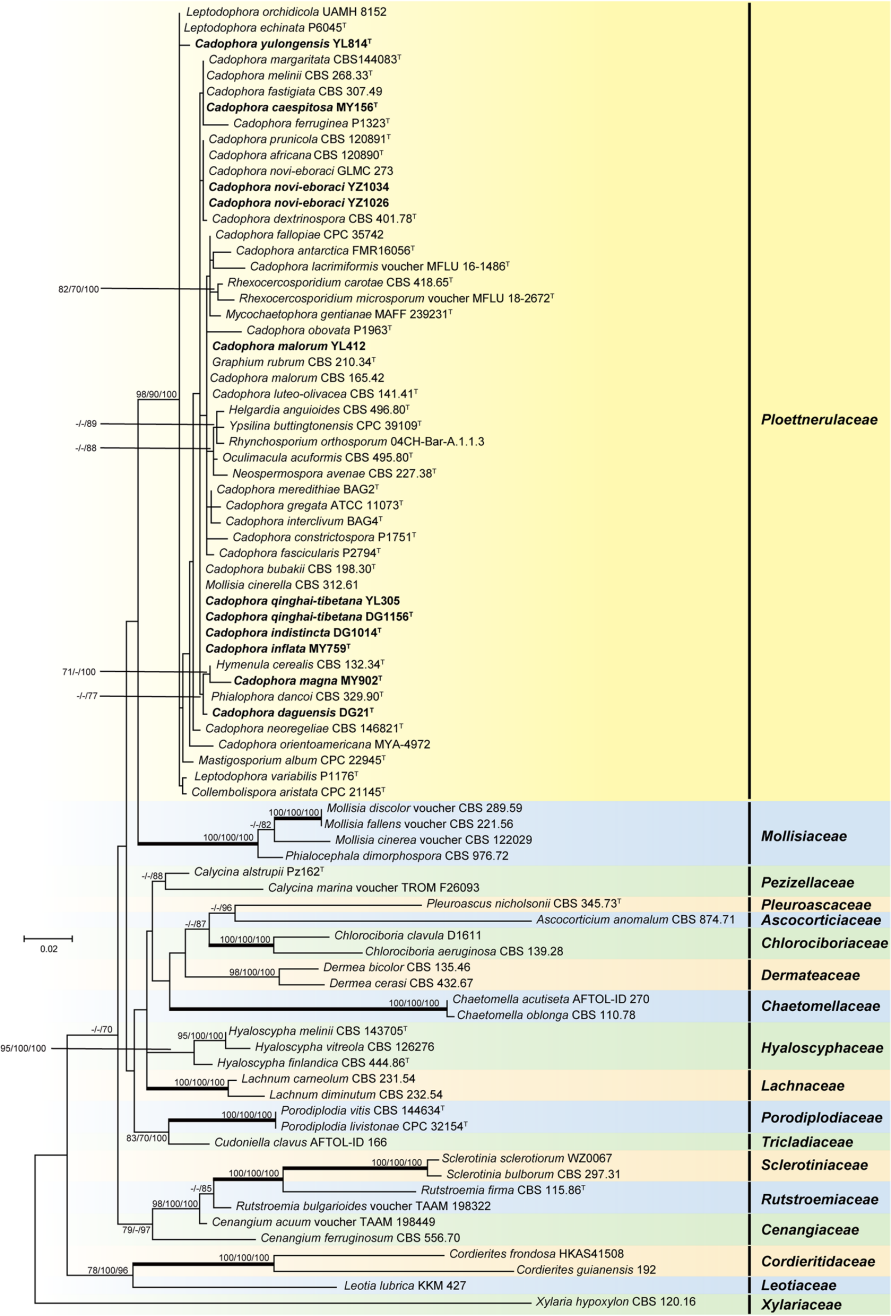
Phylogenetic tree derived from Maximum Likelihood analysis based on LSU rDNA sequences. *Xylaria hypoxylon* CBS 120.16 was used as outgroup. Sequences generated in this study are printed in bold. BP and PP values ≥ 70% are shown at nodes. Thickened branches indicate strong support with ML/MP bootstrap values = BI posterior probabilities = 100%. Ex-type cultures are marked with a superscript T. The families the isolates belong to are highlighted by colored clades, and family names are listed to the right

According to the LSU phylogenetic tree, representative *Cadophora* strains of this study (marked with bold font) and the known *Cadophora* species are interspersed with species of other genera in *Ploettnerulaceae* and form a well-supported clade (BP/BP/PP = 90/98/100, ML/MP bootstrap and BI posterior probability support values, respectively) that distinctly separate from other family members in the *Helotiales* (Fig. 3).

**Fig. 4:**
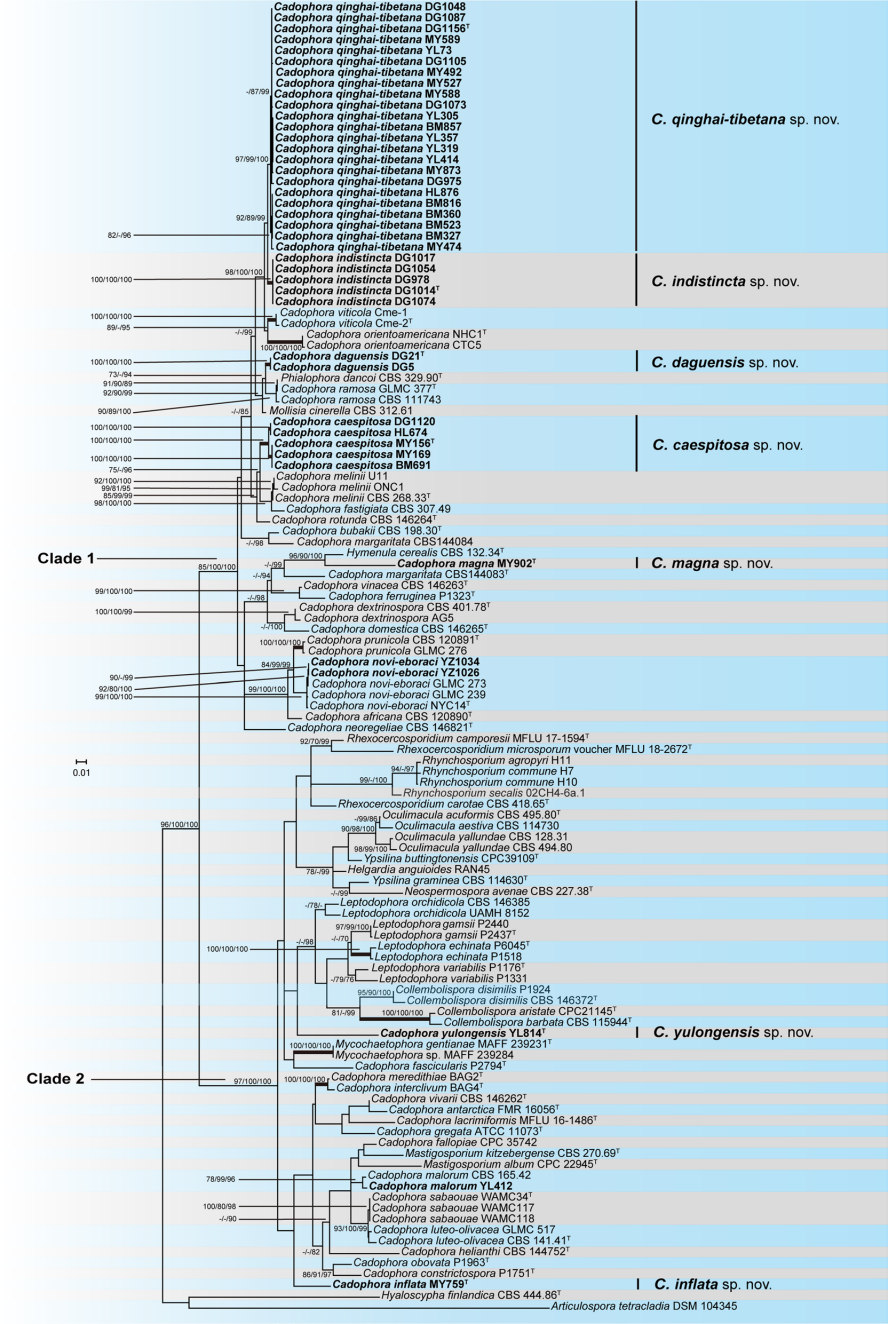
Phylogenetic tree derived from Maximum Likelihood analysis based on ITS, LSU, BT and TEF1-α combined sequence data. *Hyaloscypha finlandica* CBS 444.86^T^ and *Articulospora tetracladia* DSM 104,345 were used as outgroup. Sequences generated in this study are printed in bold. BP and PP values ≥ 70% are shown at nodes. Thickened branches indicate strong support with ML/MP bootstrap values = BI posterior probabilities = 100%. Ex-type cultures are marked with a superscript T

A multi-gene phylogenetic tree is also employed to investigate further phylogenetic relationships intra and among *Cadophora* and allied genera (Fig. 4). All the representative species cluster into two main clades with high ML/MP bootstrap or BI posterior probability support values (85/100/100, 97/100/100 respectively). In the first main clade (Clade 1), 38 isolates of this study form six distinct subclades: isolates of YZ1026 and YZ1034 cluster in a lineage including the ex-type sequences of *C. novi-eboraci* with strong branch support; although strain MY902 and the known species of *Hymenula cerealis* form a well-supported subclade, they are obviously distinguished morphologically. The placement of *H. cerealis* should also be confirmed by protein coding genes which are currently unavailable; the other four subclades group seperately from previously described species. Combined with morphological characteristics, we propose five *Cadophora* species new to science: *Cadophora caespitosa*, *C. daguensis*, *C. indistincta*, *C. magna* and *C. qinghai-tibetana*. Clade 1 also includes most of the phialidic *Cadophora* species (including the type species of the genus) and three species (*Hymenula cerealis*, *Mollisia cinerella* and *Phialophora dancoi*) currently placed in other genera. The second main clade (Clade 2) contains the remaining *Cadophora* species and most of the other *Ploettnerulaceae* members. Three isolates of this study are included in this clade: strain YL412 clusters with *C. malorum* in a well supported lineage; strain MY759 and MY814 form two distinct single strain clades and we propose them as two new species (*Cadophora inflata* and *Cadophora yulongensis*). *Cadophora* species in Clade 2 have multiform conidiogenesis modes and form lineages interspersed by other *Ploettnerulaceae* members.

## Taxonomy

**Fig. 5:**
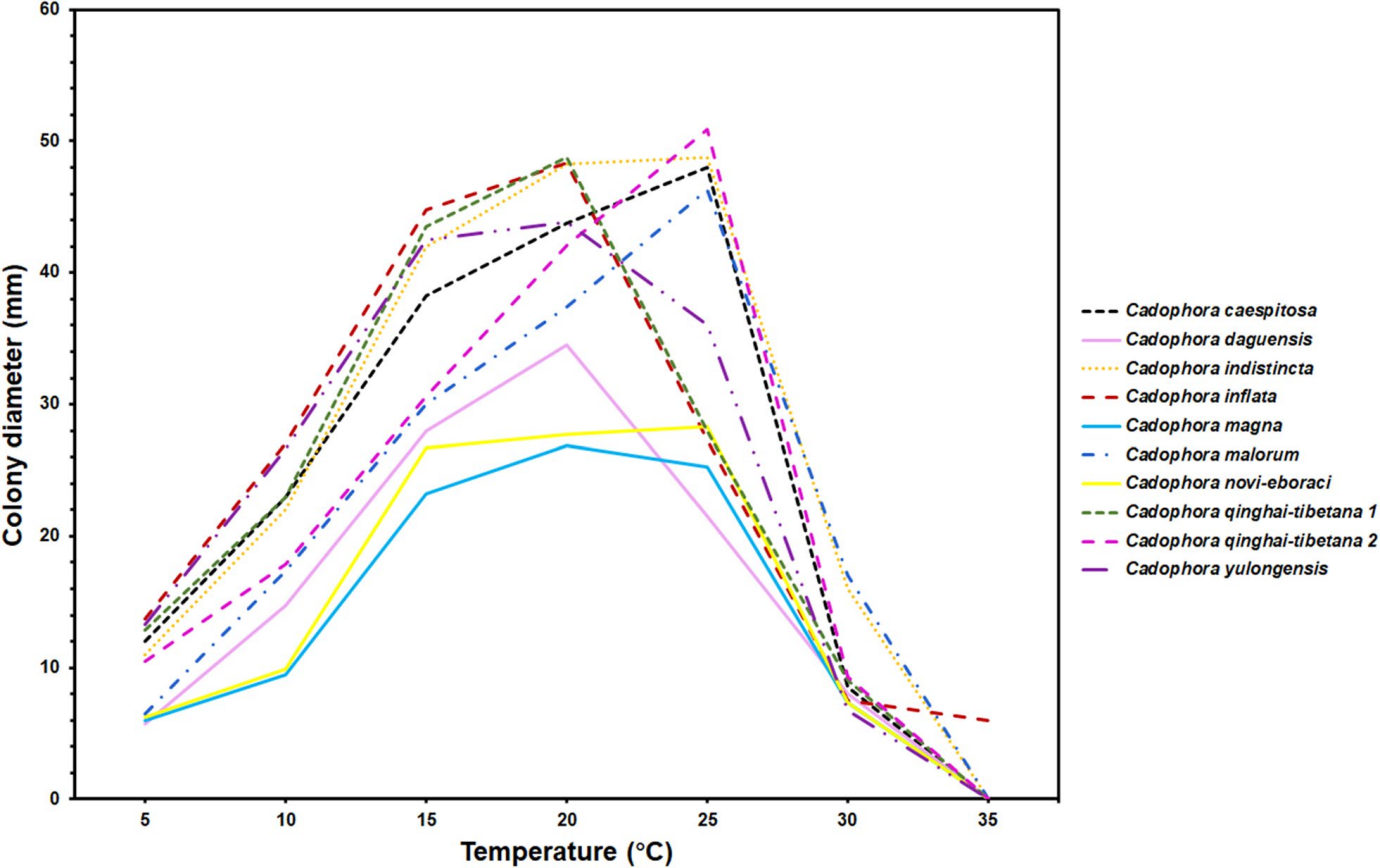
Average colony diameter of *Cadophpra caespitosa*, *C. daguensis*, *C. indistincta*, *C. inflata*, *C. magna*, *C. malorum*, *C. novi-eboraci*, *C. qinghai-tibetana* and *C. yulongensis*, assessed on PDA after 14 d growth in the dark at temperatures ranging from 5 to 35 °C, in 5 °C increments. Three PDA plates per isolate were used. (*Cadophora qinghai-tibetana* 1 and *C. qinghai-tibetana* 2 represent average colony diameters of strains with different OGTs.)

**Fig. 6:**
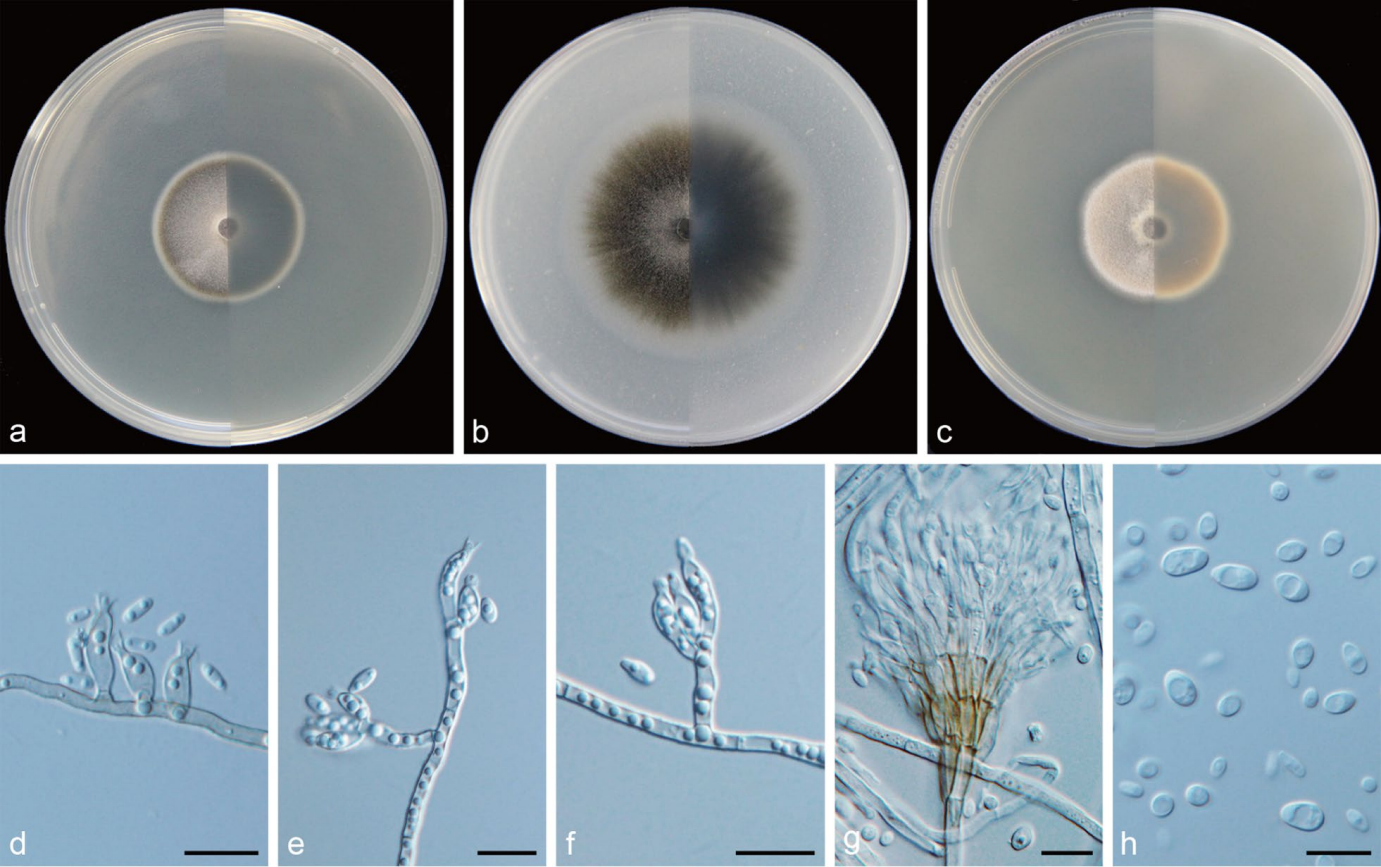
*Cadophora caespitosa* (CGMCC3.20179 – ex-type culture). **a**–**c** Front and reverse views of cultures on MEA, OA and PDA after 14 d (from left to right). **d** phialides and conidia. **e**–**f** conidiophores and conidiogenous cells. **g** fascicle of phialides. **h** conidia. Scale bars = 10 μm

**Fig. 7:**
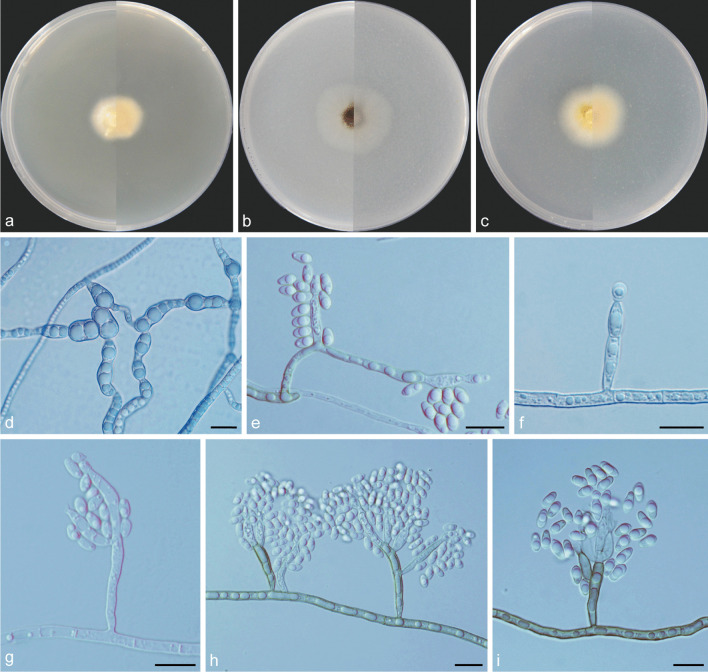
*Cadophora daguensis* (CGMCC3.20846 – ex-type culture). **a**–**c** Front and reverse views of cultures on MEA, OA and PDA after 14 d (from left to right). **d** some segments of swelled hypha. **e**–**i** conidiogenous cells and conidia. Scale bars = 10 μm

**Fig. 8:**
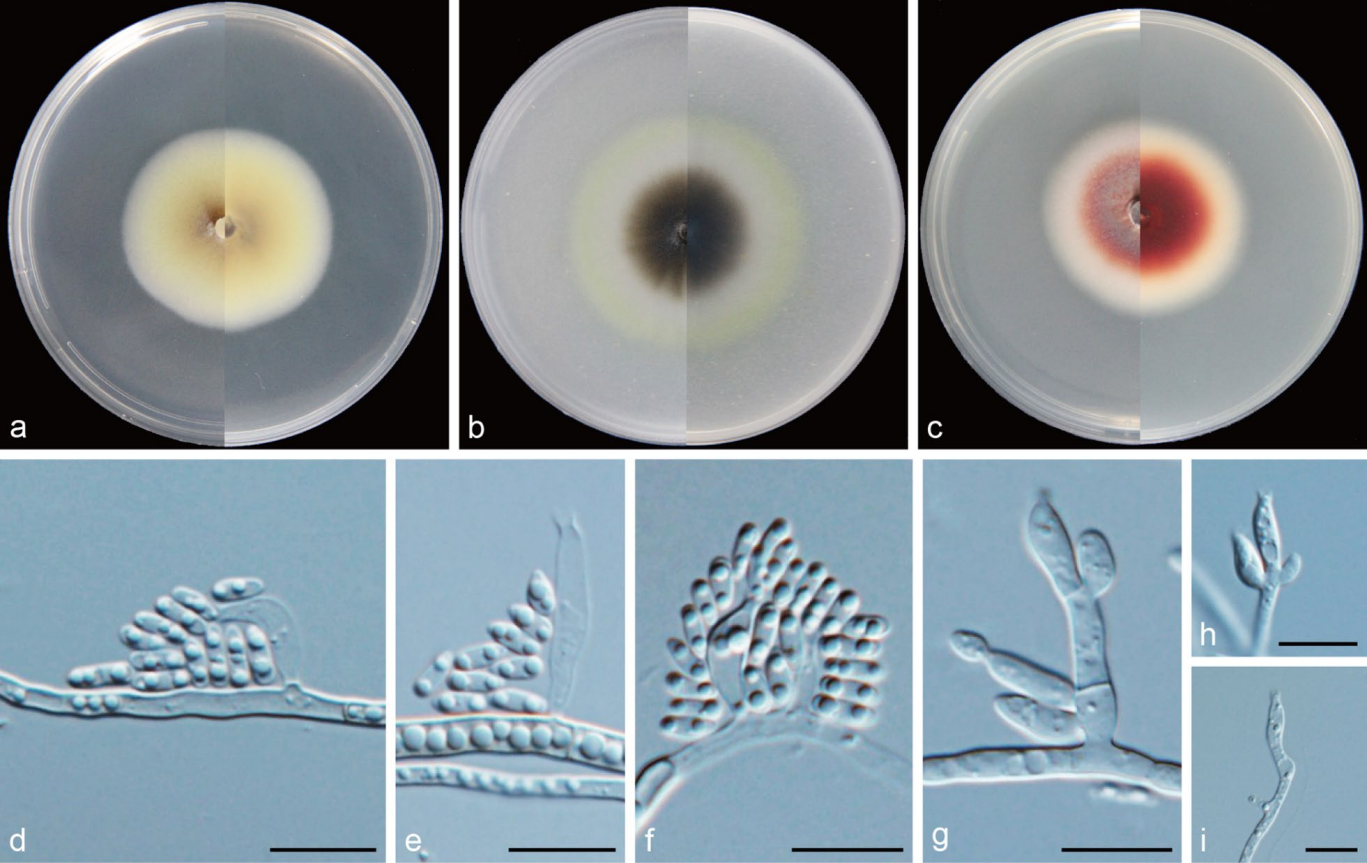
*Cadophora indistincta* (CGMCC3.20189 – ex-type culture). **a**–**c** Front and reverse views of cultures on MEA, OA and PDA after 14 d (from left to right). **d**–**f** phialides and conidia. **g-i** conidiogenous cells. Scale bars = 10 μm

**Fig. 9:**
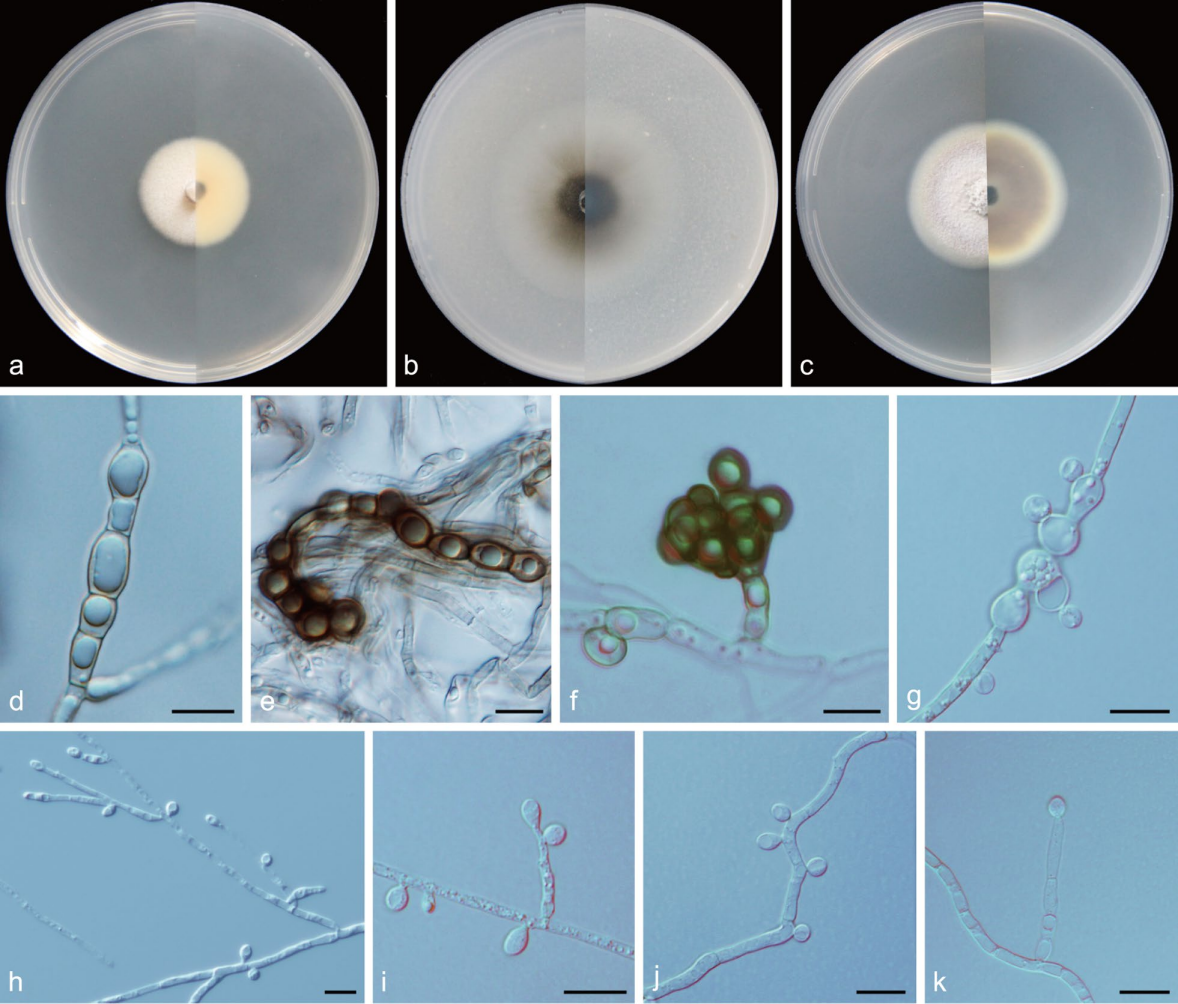
*Cadophora inflata* (CGMCC3.20186 – ex-type culture). **a**–**c** Front and reverse views of cultures on MEA, OA and PDA after 14 d (from left to right). **d** hyphal swellings. **e**–**f** microsclerotia-like bodies formed by mycelium. **g**–**k** conidia. Scale bars = 10 μm

**Fig. 10:**
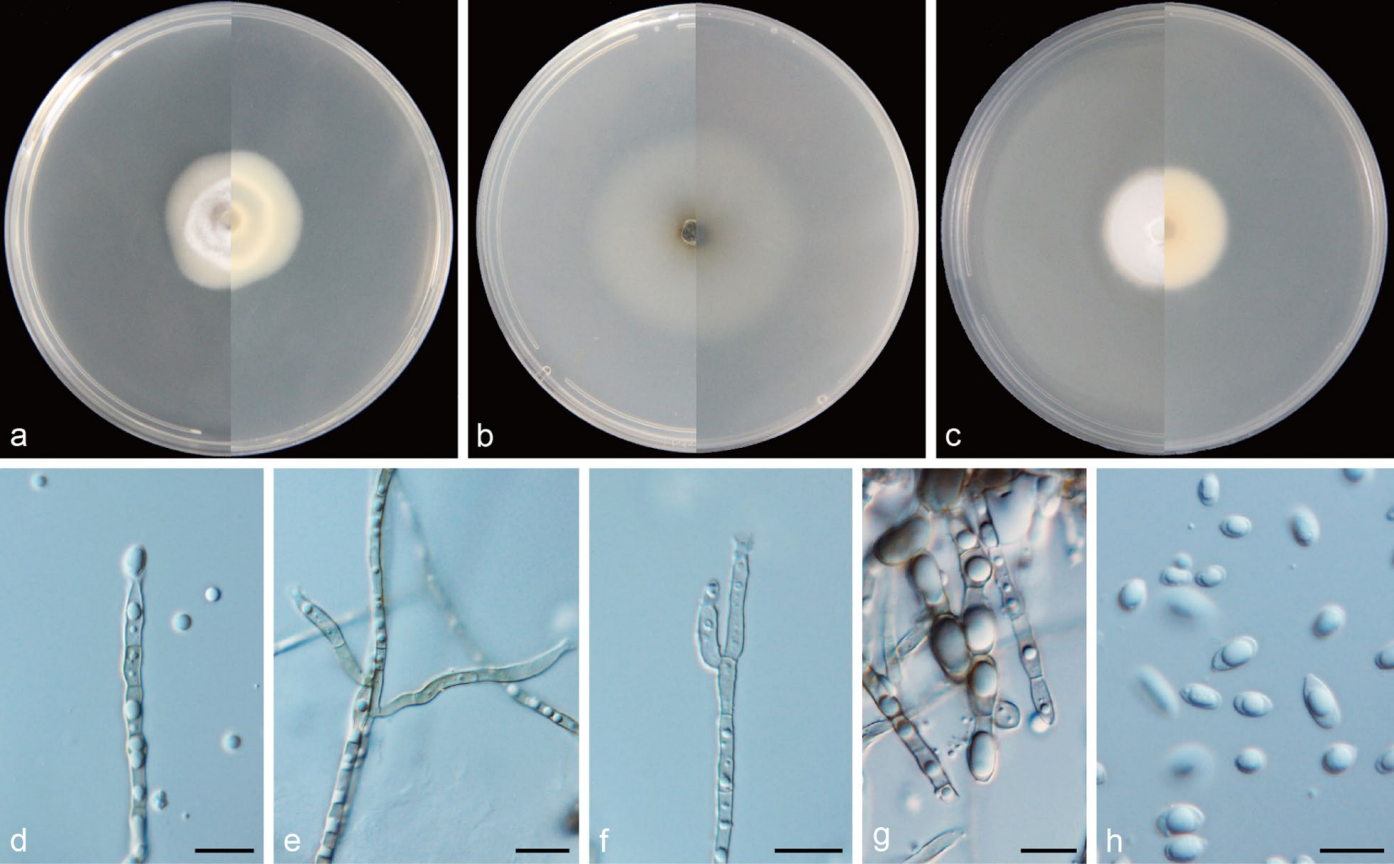
*Cadophora magna* (CGMCC3.20188 – ex-type culture). **a**–**c** Front and reverse views of cultures on MEA, OA and PDA after 14 d (from left to right). **d** single phialide producing conidium. **e**–**f** conidiophore and conidiogenous cells. **g** hyphae. **h** conidia. Scale bars = 10 μm

**Fig. 11:**
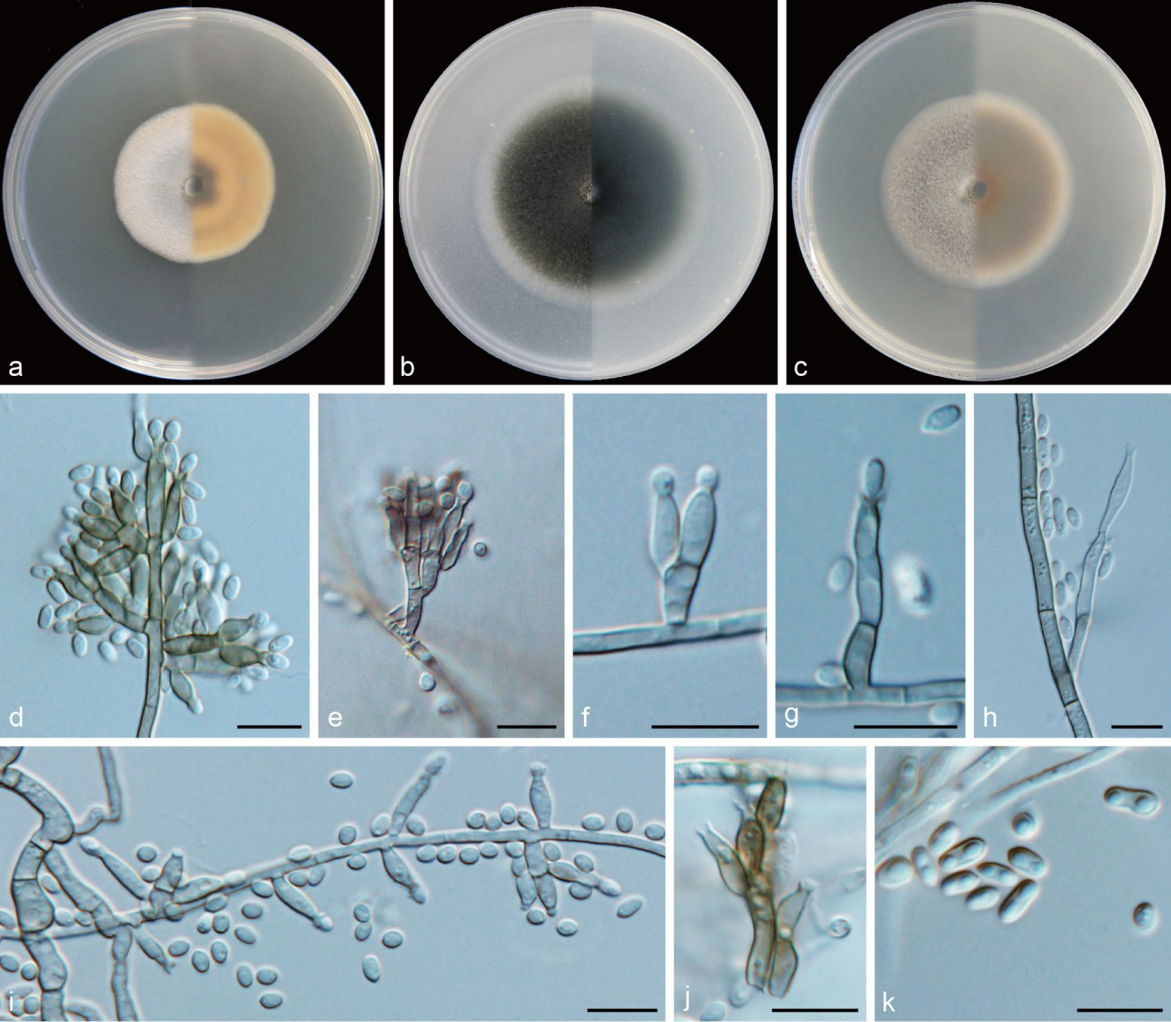
*Cadophora malorum* (CGMCC3.20184 – isolate YL412). **a**–**c** Front and reverse views of cultures on MEA, OA and PDA after 14 d (from left to right). **d**–**e** fascicle of phialides. **f**–**j** conidiophore and conidiogenous cells. **k** conidia. Scale bars = 10 μm

**Fig. 12:**
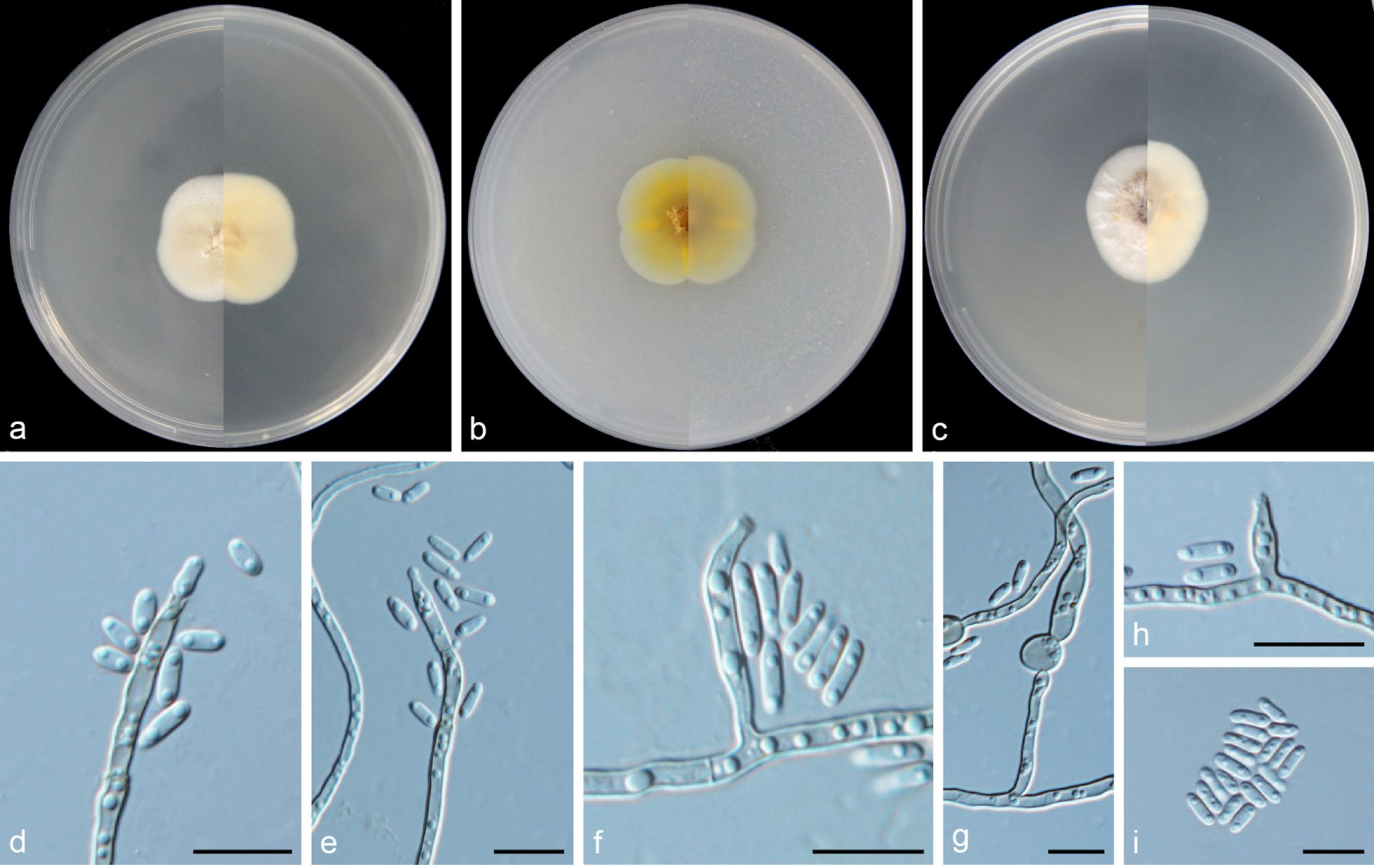
*Cadophora novi-eboraci* (CGMCC3.20190 – isolate YZ1034). **a**–**c** Front and reverse views of cultures on MEA, OA and PDA after 14 d (from left to right); d. single phialide producing conidium. **e**–**f** conidiogenous cells and conidia. **g** hyphal swellings. **h** single phialide and conidia. **i** conidia. Scale bars = 10 μm

**Fig. 13:**
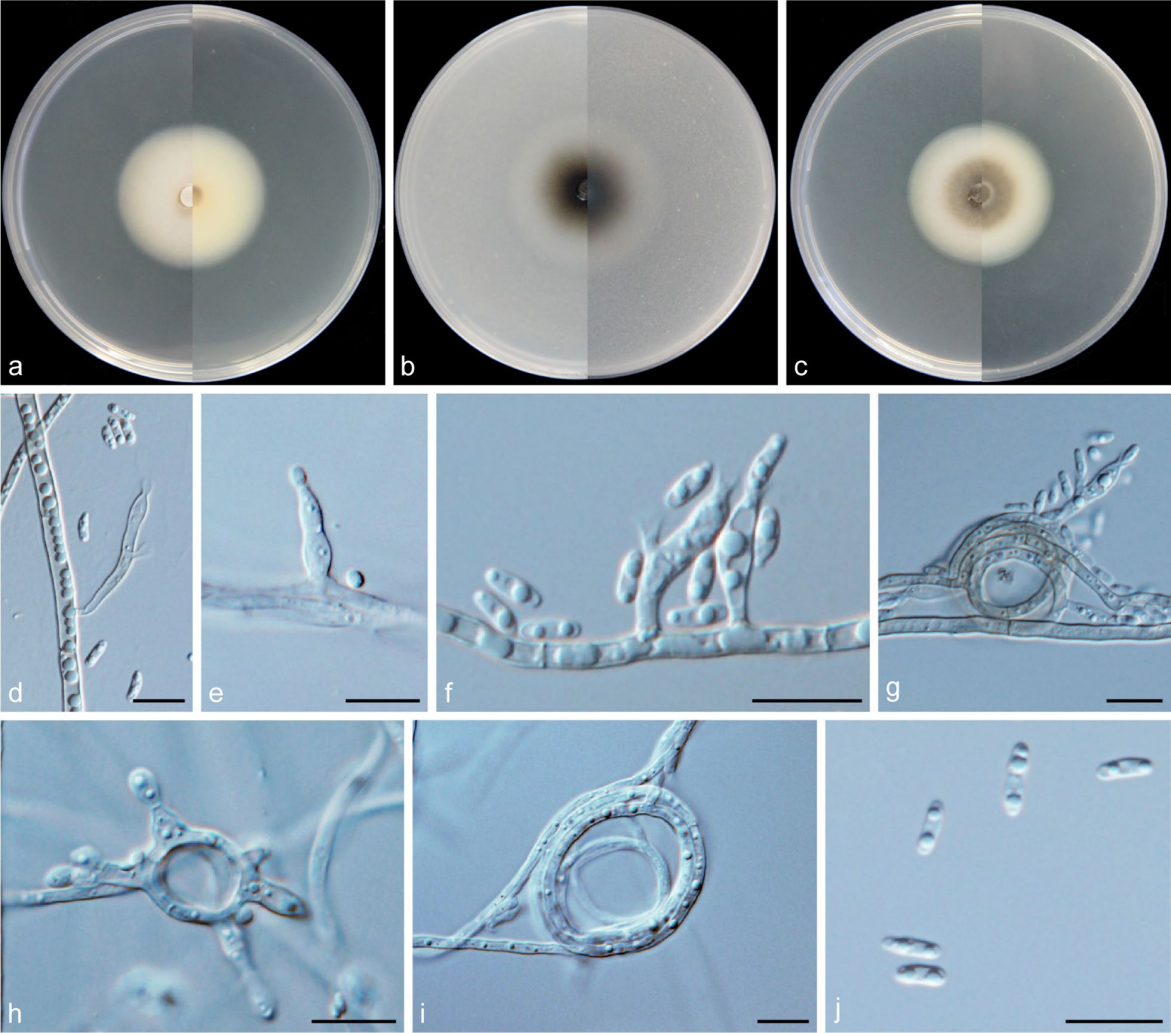
*Cadophora qinghai-tibetana* (CGMCC3.20193 – ex-type culture). **a**–**c** Front and reverse views of cultures on MEA, OA and PDA after 14 d (from left to right). **d**–**f** conidiogenous cells and conidia. **g**–**h** phialide formed on hyphal coil. **i** hyphal coil. **j** conidia. Scale bars = 10 μm

**Fig. 14:**
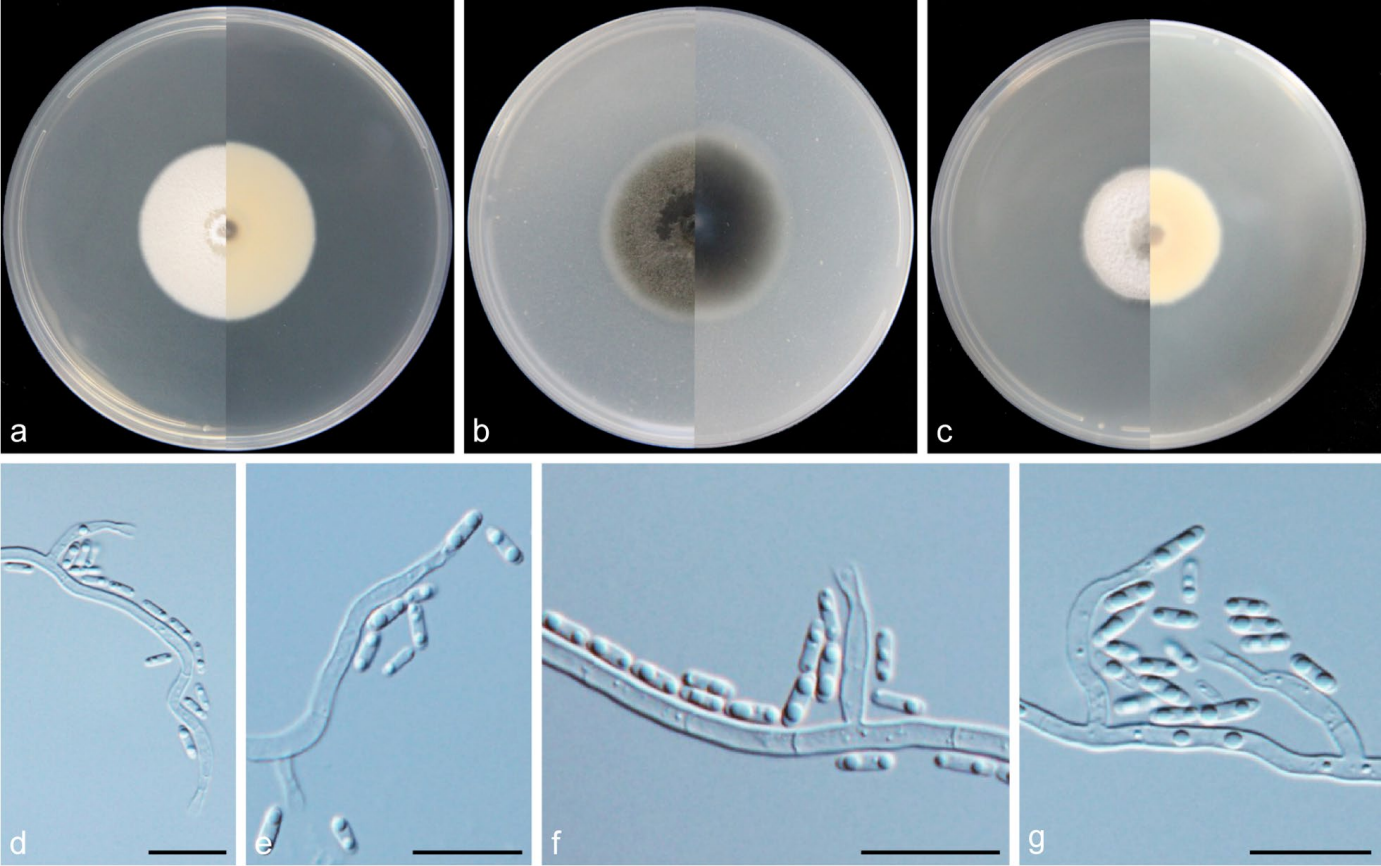
*Cadophora yulongensis* (CGMCC3.20187 – ex-type culture). **a**–**c** Front and reverse views of cultures on MEA, OA and PDA after 14 d (from left to right). **d**–**g** conidiogenous cells and conidia. Scale bars = 10 μm

- ***Cadophora caespitosa*** Q.-M. Wang, B.-Q. Zhang & M.-M. Wang, **sp. nov.**
- MycoBank No.: MB837889.
- (Fig. 6).
- *Etymology*: Tufted (Lat.: *caespitosa*). Referring to multiple phialides arranged in terminal fascicles.
- *Diagnosis*: Morphologically distinct from other *Cadophora* species in having penicillately branched heads of multiple phialides.
- *Type*: **China:** *Yunnan Province*: Mingyong Glacier, N28°27′25″ E98°45′51″, 2960 m, from water, 9 May 2017, *M.-M. Wang* (HBU20001 – holotype; MY156 = CGMCC3. 20,179 – ex-type cultures).
- *Description: Mycelium* hyaline to brown, septate, smooth-walled, branched, 1–3 μm wide. *Conidiophores* pale brown or hyaline, straight, septate, smooth, branched or unbranched, distinct, dark stipe with multiple phialides terminating in a complexly penicillately branched apex commonly observed. *Conidiogenous cells* phialidic, located laterally on fertile hyphae or arranged in complex heads, cylindrical to navicular, often constricted at the base, upper subulate, hyaline, smooth-walled, 6.5–32.3 × 2.6–3.8 μm, collarettes distinct, funnel-shaped, 1.9–3.9 μm long, opening 1.9–3.4 μm wide. *Conidia* hyaline, aseptate, smooth-walled, sporulation abundant, ovate to dacryoid or ellipsoidal, single, with both ends rounded, straight, 3.4–7.1 × 1.7–3.4 μm (mean = 5.0 ± 0.9 × 2.6 ± 0.4 μm, *n* = 30), L/W ratio = 2.0.
- *Culture characteristics* — Colonies on MEA reaching 33 mm diam after 14 d at 25 °C in the dark, on OA and PDA reaching 55 mm and 34 mm diam, respectively. OGT 25 °C and MGT 35 °C (Fig. 5). Colonies on MEA with a smooth margin, flat, grey-white, buff to light yellow at the margin, reverse olivaceous black. Colonies on OA with an entire margin, flat, greenish-black with a white margin, reverse same colours. Colonies on PDA with an entire margin, flat, hazel to yellow–brown with a white margin, reverse same colours.
- *Notes*: According to Day et al. (2012), the genera *Cadophora* and *Phialocephala* are generally distinguished by phialide complexity and conidial length, with the former producing solitary phialides and conidia longer than 4 μm, while the latter producing densely packed heads of phialides and conidia shorter than 4 μm. This newly described species is morphologically distinct from other *Cadophora* species, because it has penicillately branched heads of multiple phialides. This character is similar to species of *Phialocephala*. However, *C. caespitosa* and species of *Phialocephala* vary in conidial length. Phylogenetic analyses based on sequences of LSU and combined ITS + LSU + TUB + TEF1-α regions (Figs. 3, 4) show that *C. caespitosa* is grouped with species of *Cadophora* in the family of *Ploettnerulaceae* and forms a well-supported lineage*.*
- *Additional specimens examined*: **China:** *Sichuan Province*: Dagu Glacier, N32°14′23″ E 102°47′7″, 3610 m, from water, 1 May 2017, *M.-M. Wang* (culture DG1120 = CGMCC3.20192); Hailuogou Glacier, N29°33′10″ E101°58′10″, 3180 m, from water, 28 Apr. 2017, *M.-M. Wang* (culture HL674 = CGMCC3.20431. *Yunnan Province*: Baima Snow Mountain, N28°23′29″ E98°59′22″, 4125 m, from soil, 10 May 2017, *M.-M. Wang* (BM691 = CGMCC3.20432; Mingyong Glacier, N28°27′25″ E98°45′51″, 2960 m, from water, 9 May 2017, *M.-M. Wang* (culture MY169 = CGMCC3.20180).

- ***Cadophora daguensis*** Q.-M. Wang, B.-Q. Zhang & M.-M. Wang, **sp. nov.**
- MycoBank No.: MB837890.
- (Fig. 7).
- *Etymology*: Referring to the geographical location from where the isolates collected.
- *Diagnosis*: Morphologically distinct from the phylogenetically related species of *C. ramosa* by having lower growth rate.
- *Type*: **China:** *Sichuan Province*: Dagu Glacier, N32°14′21″ E102°47′5″, 3630 m, from soil, 1 May 2017, *M.-M. Wang* (HBU20040 – holotype; DG21 = CGMCC3.20846 – ex-type cultures).
- *Description: Mycelium* black brown or hyaline, septate, smooth-walled, branched, 1–3 μm. Mycelial cell occasionally inflated in the middle, up to 5–8 μm wide, constricted at the septae. *Conidiophores* black brown or hyaline, septate, mesotonously branched or unbranched. *Conidiogenous cells* phialidic, hyaline, smooth-walled, tapering toward the tip and slightly constricted at the base, 13.4–23.5 × 2.2–3.8 μm, collarettes distinct and funnel-shaped, 2.8–4.8 μm long, opening 2.6–3.8 μm wide. *Conidia* hyaline, aseptate, smooth-walled, with subulate tip and round base, single, straight, 4.5–7.8 × 2.1–3.2 μm (mean = 5.5 ± 0.7 × 2.7 ± 0.3 μm, *n* = 30), L/W ratio = 2.1.
- *Culture characteristics* — Colonies on MEA reaching 13 mm diam, after 14 d at 25 °C in the dark, on OA and PDA reaching 19 mm and 17 mm diam, respectively. OGT 20 °C and MGT 35 °C (Fig. 5). Colonies on MEA raised, glabrous, citrine to primrose, reverse same colours. Colonies on OA with a smooth margin, flat, olivaceous brown in the centre, light grey at the margin, reverse same colours. Colonies on PDA with a whitish margin, slight raised, pure yellow, reverse same colours.
- *Notes*: Strains of DG5 and DG21, representing *Cadophora daguensis,* form a well-supported subclade. This newly described species is phylogenetically related to *C. ramosa*, but they are obviously distinguished in colony growth rates.
- *Additional specimen examined*: **China:** *Sichuan Province*: Dagu Glacier, N32°14′21″ E102°47′5″, 3630 m, from soil, 1 May 2017, *M.-M. Wang* (culture DG5 = CGMCC3.20845).

- ***Cadophora indistincta*** Q.-M. Wang, B.-Q. Zhang & M.-M. Wang, **sp. nov.**
- MycoBank No.: MB837895.
- (Fig. 8).
- *Etymology*: Referring to the indistinct collarettes of phialides.
- *Diagnosis: Cadophora indistincta* characterized by the red coloured colony on PDA and indistinct collarettes.
- *Type*: **China:** *Sichuan Province*: Dagu Glacier, N32°8′19″ E102°56′13″, 2380 m, from water, 1 May 2017, *M.-M. Wang* (HBU20012 – holotype; DG1014 = CGMCC3.20189 – ex-type cultures).
- *Description: Mycelium* hyaline, septate, smooth-walled, branched, 1–4 μm. *Conidiophores* hyaline, septate, smooth, often solitary. *Conidiogenous cells* phialidic, located terminally or laterally, discrete, hyaline, smooth-walled, straight or curved, cylindrical to navicular, often inflated in the middle and constricted at the base, 5.3–31.4 × 1.6–3.7 μm, collarettes often indistinct. *Conidia* hyaline, aseptate, smooth-walled, cylindrical to oblong, 4.7–7.5 × 1.6–2.5 μm (mean = 5.5 ± 0.7 × 2.2 ± 0.2 μm, *n* = 30), L/W ratio = 2.5.
- *Culture characteristics* — Colonies on MEA reaching 45 mm diam, after 14 d at 25 °C in the dark, on OA and PDA reaching 49 mm and 44 mm diam, respectively. OGT 25 °C and MGT 35 °C (Fig. 5). Colonies on MEA flat, primrose to pale citrine, white at the margin, reverse same colours. Colonies on OA with a yellow margin, surface black-brown, aerial mycelium sparse, reverse same colours. Colonies on PDA with a distinct and smooth margin, flat, grey to red, white at the edge, reverse dark-red.
- *Notes*: *Cadophora indistincta* is phylogenetically related to *C*. *qinghai-tibetana* (Fig. 4), but they are especially different in colours of colony on PDA and the length of collarettes (Figs. 8, 13). *Cadophora indistincta* produces red coloured colony on PDA and this is also a distinct character different from other *Cadophora* species except *C. ferruginea,* but the colour of the colony produced by *C. ferruginea* is rust red and darker than that of *C. indistincta*.
- *Additional specimens examined*: **China:** *Sichuan Province*: Dagu Glacier, N32°8′19″ E102°56′13″, 2380 m, from soil, 1 May 2017, *M.-M. Wang* (culture DG978 = CGMCC3.20233; DG1074 = CGMCC3.20196); N32°15′38″ E102°48′15″, 3510 m, from soil, 1 May 2017, *M.-M. Wang* (culture DG1017 = CGMCC3.20195); N32°14′23″ E102°47′7″, 3610 m, from water, 1 May 2017, *M.-M. Wang* (cculture DG1054 = CGMCC3.20234).

- ***Cadophora inflata*** Q.-M. Wang, B.-Q. Zhang & M.-M. Wang, **sp. nov.**
- MycoBank No.: MB837892.
- (Fig. 9).
- *Etymology*: Referring to the characteristics of the inflated hyphae.
- *Diagnosis*: Morphologically distinct from other *Cadophora* species by producing multiple chlamydospores and single holoblastic conidia attached to the hyphae with short conidiophores.
- *Type*: **China:** *Yunnan Province*: Mingyong Glacier, N28°27′24″ E98°45′51″, 2976 m, from soil, 9 May 2017, *M.-M. Wang* (HBU20009 – holotype; MY759 = CGMCC3.20186 – ex-type cultures).
- *Description: Mycelium* olivaceous or hyaline, septate, branched, smooth-walled, 2–4 μm wide. Hyphal cells often strongly inflated, up to 6–10 μm wide, form chains or microsclerotia-like bodies. *Conidiophores* very short or highly reduced. *Conidiogenous cells* holoblastic. *Conidia* hyaline, attached to mycelium, located laterally or terminally, smooth-walled, globular or spathulate, solitary, 2.9–7.1 × 3.0–4.4 μm (mean = 3.9 ± 0.8 × 3.7 ± 0.4 μm, *n* = 30), L/W ratio = 1.1.
- *Culture characteristics* — Colonies on MEA reaching 28 mm diam, after 14 d at 25 °C in the dark, on OA and PDA reaching 47 mm and 37 mm diam, respectively. OGT 25 °C and MGT over 35 °C (Fig. 5). Colonies on MEA, with an entire margin, flat, white, lacking aerial mycelium, reverse same colours. Colonies on OA with a smooth margin, flat, black in the center, olivaceous to white from middle to edge, reverse same colours. Colonies on PDA with a smooth margin, felty, grey, pale yellow at the margin, reverse grey-brown with a pale buff to white margin.
- *Notes*: *Cadophora inflata* is characterized by chains or microsclerotia-like inflated cells that are similar to *Leptodophora gamsii* and *L. echinata* which were first described as *C. gamsii* and *C. echinata* (Maciá-Vicente et al. 2020). The original authors interpreted these structures as holoblastic conidia but they may just as well described as inflated hyphal segments with dormancy functions. Our newly described species failed to produce conidia on MEA, OA, and PDA media. We also tried other methods such as treating the cultures with H_2_O_2_ or culturing the isolates on pine needle medium before a slide culture technique was used. *Cadopohra inflata* produces globose or ellipsoidal conidia attached directly to the hyphae with very short conidiophores that resemble those of *Leptodophora orchidicola*, which has been transferred from *Cadophora* to *Leptodophora* (Koukol & Maciá-Vicente, 2022). Thus, we presume that the inflated hyphal cells are really just chlamydospores.

- ***Cadophora magna*** Q.-M. Wang, B.-Q. Zhang & M.-M. Wang, **sp. nov.**
- MycoBank No.: MB837893.
- (Fig. 10).
- *Etymology*: Big (Lat.: *magna*). Referring to the comparatively large conidia.
- *Diagnosis*: *Cadophora magna* is distinct in producing the large conidia and strongly inflated hyphal cells.
- *Type*: **China:** *Yunnan Province*: Mingyong Glacier, N28°27′24″ E98°45′51″, 2976 m, from soil, 9 May 2017, *M.-M. Wang* (HBU20011 – holotype; MY902 = CGMCC3.20188 – ex-type cultures).
- *Description: Mycelium* hyaline to dark brown, septate, branched, smooth-walled, 1–3 μm, hyphal cells often strongly inflated, variable in shape. *Conidiophores* brown, smooth-walled, often reduced to conidiogenous cells. *Conidiogenous cells* phialidic, mostly single, arranged terminally or laterally on the hyphae, cylindrical to navicular, apex wedge, base truncate, smooth-walled, straight or slightly curved, 12.7–20.3 × 2.8–3.8 μm, collarettes funnel-shaped, 1.9–3.0 μm long, opening 2.8–2.9 μm wide. *Conidia* hyaline, aseptate, smooth-walled, ovoidal or dacryoid to ellipsoidal, upper wedge-shaped, base round, single, straight, 5.2–9.4 × 3.0–4.7 μm (mean = 7.3 ± 0.9 × 3.7 ± 0. 4 μm, *n* = 30), L/W ratio = 2.0.
- *Culture characteristics* — Colonies on MEA reaching 30 mm diam after 14 d at 25 °C in the dark, on OA and PDA reaching 41 mm and 29 mm diam, respectively. OGT 20 °C and MGT 35 °C (Fig. 5). Colonies on MEA white, margin covered with white and velvety aerial mycelium, reverse white. Colonies on OA with a smooth margin, flat, whitish, pale olivaceous in the centre, reverse same colours. Colonies on PDA white, reverse same colours.
- *Notes*: *Cadophora magna* is currently only known from a single isolate (MY902) from soil samples of Mingyong Glacier and is morphologically distinct from other *Cadophora* species in the huge single conidia. In the newly described species, both *C. magna* and *C. inflata* produce strongly inflated hyphae cells, but the hyphae cells of *C. inflata* are often thick-walled and form tuft-like bodies. *C. magna* is phylogenetically related to *Hymenula cerealis,* but they are obviously distinguished morphologically, as the latter often produces short chains of spores as well as spores enveloped in a mucus drop (Nisikado et al. 1934). Besides, the placement of *H. cerealis* should also be confirmed by more molecular data which are currently unavailable.

- ***Cadophora malorum*** (Kidd & Beaumont) W. Gams, *Stud. Mycol.* 45: 188 (2000).
- (Fig. 11).
- *Description: Mycelium* brown-black, septate, smooth-walled, branched, 2–3 μm. *Conidiophores* brown-black, septate, smooth. *Conidiogenous cells* phialidic, often forming clusters, terminally or laterally on the hyphae, smooth-walled, straight, ampulliform, often 9.5–16.0 × 2.9–3.5 μm, collarettes distinct, collarettes short tubular to funnel-shaped, 1.1–2.0 μm long, opening 1.6–1.9 μm wide. *Conidia* fuscous, aseptate, smooth-walled, ellipsoidal to elongate-ellipsoidal or subglobose, single, straight, 2.7–4.7 × 1.9–3.4 μm (mean = 3.7 ± 0.5 × 2.5 ± 0.4 μm, *n* = 30), L/W ratio = 1.5.
- *Culture characteristics* — Colonies on MEA reaching 41 mm diam, after 14 d at 25 °C in the dark, on OA and PDA reaching 60 mm and 48 mm diam, respectively. OGT 25 °C and MGT 35 °C (Fig. 5). Colonies on MEA with a weakly undulate margin, brown-grey to yellow–brown, reverse same colours. Colonies on OA with a distinct and white margin, olivaceous to dull green, reverse same colours. Colonies on PDA with a distinct margin, felty, brown, reverse yellow–brown.
- *Notes: Cadophora malorum* is a very common *Cadophora* species and has often been isolated as saprobes or pathogens worldwide (Blanchette et al. 2010; Gramaje et al. 2011; Sugar and Spotts 1992). Strain YL412 was isolated from soil samples collected from Yulong Snow Mountain and the morphological characteristics are similar with the description of the type (Gams, 2000).
- *Specimen examined*: **China:** *Yunnan Province*: Yulong Snow Mountain, N27°11′17″ E100°22′43″, 3362 m, from soil, 7 May 2017, *M.-M. Wang* (culture YL412 = CGMCC3.20184).

- ***Cadophora novi-eboraci*** Travadon et al*., Fungal Biol.* 119: 61 (2015).
- (Fig. 12).
- *Description: Mycelium* hyaline to brown, septate, smooth-walled, branched, 1–3 μm. *Conidiophores* hyaline, aseptate, smooth, often solitary. *Conidiogenous cells* phialidic, terminally or laterally on the hyphae, discrete conidiogenous cells hyaline, smooth-walled, curved or straight, cylindrical to navicular, 6.2–19.9 × 2.4–3.0 μm, collarettes short, tubular, 1.0–1.9 μm long, opening 1.4–1.8 μm wide. *Conidia* hyaline, aseptate, smooth-walled, elongate-ellipsoidal to cylindrical, straight, 3.9–8.3 × 1.8–2.7 μm (mean = 5.8 ± 1.0 × 2.3 ± 0.3 μm, *n* = 30), L/W ratio = 2.6.
- *Culture characteristics* — Colonies on MEA reaching 29 mm diam, after 14 d at 25 °C in the dark, on OA and PDA reaching 26 mm and 28 mm diam, respectively. OGT 25 °C and MGT 35 °C (Fig. 5). Colonies on MEA with an undulate margin, surface white, reverse same colours. Colonies on OA with a distinct margin, flat, citrine to pure yellow, white at edge, reverse same colours. Colonies on PDA with a distinct margin, raised, white to whitish, sometimes covered by floccose aerial mycelium, reverse same colours.
- *Notes*: *Cadophora novi-eboraci* was originally described from decaying wood of Grapevine in North America mainly based on phylogenetic analyses of three nuclear loci (ITS, TUB and TEF1-α) (Travadon et al. 2015). It has also been isolated from *Prunus* wood and freshwater (Bien and Damm 2020; Lim et al. 2021). Strains observed in this study were isolated from soil samples of the Yanzigou Glacier in China.
- *Specimens examined*: **China:** *Sichuan Province*: Yanzigou Glacier, N29°41′58″ E102°0′7″, 2620 m, from soil, 29 Apr. 2017, *M.-M. Wang* (culture YZ1026 = CGMCC3.20434; YZ1034 = CGMCC3.20190).

- ***Cadophora qinghai-tibetana*** Q.-M. Wang, B.-Q. Zhang & M.-M. Wang, **sp. nov.**
- MycoBank No.: MB837896.
- (Fig. 13).
- *Etymology*: Referring to the geographical location from where the type strain was collected.
- *Diagnosis*: Morphologically distinguished from the phylogenetically related species of *C. indistincta* in colony colours and the length of collarettes.
- *Type*: **China:** *Sichuan Province*: Dagu Glacier, N32°8′19″ E102°56′13″, 2380 m, from soil, 1 May 2017, *M.-M. Wang* (HBU20019 – holotype; DG1156 = CGMCC3.20193 – ex-type cultures).
- *Description: Mycelium* hyaline or brown-black, septate, smooth-walled, branched, 2–4 μm, often forming coils up to 34.9 μm diam. *Conidiophores* hyaline, smooth, frequently reduced to conidiogenous cells. *Conidiogenous cells* phialidic, laterally on the hyphae or hyphae coils, single or in groups of two or three, the mesotonously branched ones often reduced to mere openings with collarettes formed directly on conidiophores, cylindrical or navicular, inflated in the middle and attenuated at the base, hyaline or fuscous, smooth-walled, straight or curved, 6.8–19.9 × 2.0–3.9 μm, collarettes funnel-shaped or absent, 1.6–2.5 μm long, opening 1.6–2.7 μm wide. *Sporulation abundant*, conidia hyaline, aseptate, smooth-walled, cylindrical to elongate-ellipsoidal, 5.0–7.3 × 1.7–2.7 μm (mean = 6.0 ± 0.7 × 2.1 ± 0.2 μm, *n* = 30), L/W ratio = 2.8.
- *Culture characteristics* — Colonies on MEA reaching 19 mm diam after 14 d at 25 °C in the dark, on OA reaching 31 mm and 18 mm diam, respectively. Colonies on MEA with a distinct margin, flat, colony surface buff, reverse same colours. Colonies on OA with a smooth margin, flat, surface olivaceous black, whitish at the margin, reverse same colours. Colonies on PDA with a distinct and regular margin, aerial mycelium sparse, grey in the centre, buff to whitish at the margin, reverse same colours.
- *Notes*: More than half of the isolates in this study were identified as *Cadophora qinghai-tibetana* and these were isolated from soil and water samples of Yulong Glacier, Mingyong Glacier, Baima Snow Mountain in Yunnan Province and Dagu Glacier in Sichuan Province. Strains of YL73 (from Yulong Snow Mountain), DG1048, DG1073, DG1087, DG1105 and DG1156 (from Dagu Glacier), MY527, MY588, MY589 and MY873 (from Mingyong Glacier) have optimum growth temperature of 20 °C while the others have optimum growth temperature at 25 °C. *Cadophora qinghai-tibetana* has typical phialidic conidiogenesis and produces cylindrical to ellipsoidal conidia that are common in many *Cadophora* species, but morphologically distincts from the phylogenetically related species of *C. indistincta* in colony colours and the length of collarettes.
- *Additional specimens examined*: **China:** *Sichuan Province*: Dagu Glacier, N32°13′14″ E102°45′29″, 4850 m, from soil, 1 May 2017, *M.-M. Wang* (culture DG975 = CGMCC3.20232); N32°8′19″ E102°56′13″, 2380 m, from soil, 1 May 2017, *M.-M. Wang* (culture DG1048 = CGMCC3.20191; DG1073 = CGMCC3.20235; DG1087 = CGMCC3.20236; DG1105 = CGMCC3.20197). *Sichuan Province*: Hailuogou Glacier, N29°34′8″ E101°59′36″, 3180 m, from soil, 28 Apr. 2017, *M.-M. Wang* (culture HL876 = CGMCC3.20437). *Yunnan Province*: Baima Snow Mountain, N29°23′1″ E99°0′20″, 4366 m, from soil, 10 May 2017, *M.-M. Wang* (culture BM327 = CGMCC3.20181; BM360 = CGMCC3.20183; BM523 = CGMCC3.20230; BM816 = CGMCC3.20436); N28°22′59″ E99°0′31″, 4343 m, from soil, 10 May 2017, *M.-M. Wang* (culture BM857 = CGMCC3.20433; Mingyong Glacier, N28°27′27″ E98°45′49″, 2976 m, from soil, 9 May 2017, *M.-M. Wang* (culture MY474 = CGMCC3.20185); N28°27′28″ E98°45′43″, 3067 m, from soil, 9 May 2017, *M.-M. Wang* (culture MY492 = CGMCC3.20847; MY527 = CGMCC3.20848; MY588 = CGMCC3.20849; MY589 = CGMCC3.20850; MY873 = CGMCC3.20231); Yulong Snow Mountain, N27°10′55″ E100°19′87″, 4531 m, from soil, 7 May 2017, *M.-M. Wang* (culture YL73 = CGMCC3.20228); N27°11′17″ E100°22′43″, 3362 m, from water, 7 May 2017, *M.-M. Wang* (culture YL305 = CGMCC3.20435; YL319 = CGMCC3.20229; YL357 = CGMCC3.20182; YL414 = CGMCC3.20194).

- ***Cadophora yulongensis*** Q.-M. Wang, B.-Q. Zhang & M.-M. Wang, **sp. nov.**
- MycoBank No.: MB837894.
- (Fig. 14).
- *Etymology*: Referring to Yulong Snow Mountain, the geographic origin of the type strain.
- *Diagnosis*: Morphologically distinguished from the phylogenetically related species of *Leptodophora* and *Collembolispora* in conidiogenesis and conidial shapes.
- *Type*: **China:** *Yunnan Province*: Yulong Snow Mountain, N27°10′52″ E100°19′84″, 4531 m, from soil, 7 May 2017, *M.-M. Wang* (HBU20010 – holotype; YL814 = CGMCC3.20187 – ex-type cultures).
- *Description: Mycelium* hyaline, septate, smooth-walled, branched, 1–3 μm wide. *Conidiophores* hyaline, smooth, often reduced to conidiogenous cells. *Conidiogenous cells* phialidic, located laterally or terminally, cylindrical or navicular, apex wedge, base truncate, hyaline, smooth-walled, straight or bent, 11.4–25.5 × 1.6–3.1 μm, collarettes evident, 2.1–4.5 μm long, opening 1.6–2.5 μm wide. *Conidia* hyaline, aseptate, smooth-walled, cylindrical, sporulation abundant, single, straight, 4.5–6.9 × 1.4–2.5 μm (mean = 5.5 ± 0.6 × 1.9 ± 0.3 μm, *n* = 30), L/W ratio = 2.9.
- *Culture characteristics* — Colonies on MEA reaching 36 mm diam, after 14 d at 25 °C in the dark, on OA and PDA reaching 38 mm and 28 mm diam, respectively. Colonies on MEA pale pink to whitish, white at the margin, reverse same colours. Colonies on OA black-grey with light grey margin, reverse same colours. Colonies on PDA felty, grey to pale grey, reverse pale yellow.
- *Notes*: *Cadophora yulongensis* failed to produce conidia when cultured on MEA, OA, and PDA media. Other efforts including pine needle medium culturing and H_2_O_2_ treatment (Xu et al. 2009) also failed to induce sporulation until we used a slide culture technique. In the multigene phylogenetic tree (Fig. 4), *C. yulongensis* is closely related to lineages formed by species of *Leptodophora* and *Collembolispora*. The genus *Leptodophora* is currently proposed to accommodate species firstly described as *Cadophora*. All *Leptodophora* species produce rarely seceding conidia and the conidial morphology differs markedly (Koukol & Maciá-Vicente, 2022). Species of *Collembolispora* often produce multicellular macroconidia with appendages and a synasexual morph of phialides (Marvanová et al. 2003). The newly described species is characterized by long cylindrical phialides and cylindrical conidia with comparatively high conidium length/width ratio (2.9).

## Discussion

Species of *Cadophora* have been reported from different locations worldwide, mainly as plant pathogens or root colonizers from northern temperate regions or decomposers from the cold Arctic and Antarctic environments (Blanchette et al. 2004, 2010, 2016, 2021; Duran et al. 2019; Gramaje et al. 2011; Maciá-Vicente et al. 2020; Travadon et al. 2015; Walsh et al. 2018). The Qinghai-Tibet Plateau, which is also called “the third pole”, is located in the southwest of China and is the highest and largest low-latitude region with permafrost in the world. The unique geographic location of high elevation and low latitude makes the Qinghai-Tibet Plateau a unique alpine ecosystem that is more sensitive to changes of climate and surface conditions (Cheng 1998). Warm, moist air from the Indian Ocean flows up the valleys and is then blocked by huge mountains, leading to abundant rainfall in the southeast range of the plateau. Large numbers of marine glaciers are formed in this area (Shi et al. 1964). During the investigation of cold-adapted fungi from marine glaciers in the Qinghai-Tibet Plateau in 2017, 1208 fungal strains were isolated and identified based on preliminary analyses of generated ITS sequences. Forty-one isolates belonging to *Cadophora*, one of the three most commonly encountered genera (*Cadophora*, *Geomyces* and *Pseudogymnoascus*; the latter two will be discussed in another paper) were studied in detail. Our results revealed seven *Cadophora* species, represented by 38 isolates, new to science and three isolates identified to two known species (*C. malorum* and *C. novi-eboraci*).

Because of the limited discriminating morphological characteristics existing among *Cadophora* and related genera, the genus has suffered taxonomic flux since the beginning of its establishment. DNA sequences have provided critical information for species delimitation. Some *Cadophora* species with multiform morphological characters deviate from the original generic concept, such as *C. antarctica*, *C. fallopiae, C. fascicularis,* and *C. obovata,* have been described mainly based on molecular data (Crous et al. 2017, 2020; Maciá-Vicente et al. 2020). Day et al. (2012) tried to find some consistencies between morphological characteristics and phylogenetic relationships in *Cadophora* and the related genera. They hypothesized that the ancestral state for these taxa was the production of sclerotium-like heads of multiple phialides and clades derived from phialide arrangements agreed with those generated from rDNA ITS sequence analyses. Although ITS is useful for most fungal species identification, it often fails to discriminate species or even results in misleading information in this group. For example, according to the ITS analyses, *Cadophora malorum* CBS 165.42 is nested within the *Cadophora luteo-olivacea* clade, but in the TEF tree, *C. malorum* CBS 165.42 is strongly supported as the sister group to *C. luteo-olivacea* (Travadon et al. 2015) and the RPB1 gene can also resolve species relationships between *C. meredithiae* and *C. interclivum* better than the ITS (Walsh et al. 2018); *C. microspora* only known from the sexual morph, was first identified based on ITS and morphological characteristics by Ekanayaka et al. (2019), but in recent studies, it was transferred to *Rhexocercosporidium* based on LSU and ITS analyses (Hyde et al. 2020). With more genes and species included, Maciá-Vicente et al. (2020) provided a more comprehensive overview about the ecology, morphology and phylogeny of *Cadophora*. Their results show that the genus is apparently paraphyletic and encompasses a broad spectrum of morphologies and life-styles. They tended to split the genus into three genera: one included those referred to as ‘*Cadophora s. str.* species’ that evolved from an ancestor with phialidic conidiogenesis; the second included species like *C. interclivum*, *C. meredithiae*, *C. luteo-olivacea*, *C. malorum*, and *C. helianthi* that produced conidia phialidically but are clustered in a separate clade; the third genus should take the name of *Collembolispora* including *Cadophora* species with holoblastic conidiogenesis. But this drastic restructuring still needs to be confirmed. Our multi-gene phylogenetic analyses confirmed paraphyly in *Cadophora* and all the species involved are clustered into two main clades (Fig. 4). Clade 1 comprised 21 *Cadophora* species (including five newly described in this study and the type species of the genus) and three species belonging to other genera (*Hymenula cerealis*, *Mollisia cinerella*, and *Phialophora dancoi*). This clade was similar to the ‘*Cadophora s. str*.’ clade defined by Maciá-Vicente et al. (2020), just with more species involved in our study. Although all species in Clade 1 have phialidic conidiogenesis, it is somewhat arbitrary to combine *P. dancoi*, *M. cinerella*, and *H. cerealis* into *Cadophora* at present, as we have just assembled the ITS data sets of these three species to maximize taxon coverage and more exact morphological examinations also need to be done for these fungi. Clade 2 includes most members of *Ploettnerulaceae* and the remaining *Cadophora* species. *Cadophora constrictospora*, *C. gregata*, *C. helianthi, C. interclivum*, *C. luteo-olivacea*, *C. malorum*, *C. meredithiae*, *C. sabaouae,* and *C. vivarii* which have phialidic conidiogenesis cluster with species including *C. antarctica*, *C. fallopiae*, *C. inflata, C. obovata,* and two species of *Mastigosporium* which produce conidia with putative enteroblastic or holoblastic conidogenesis. Specimens of *C. lacrimiformis* only known by the sexual morph is also in this lineage; *Leptodophora gamsii*, *L. echinata*, *L. orchidicola*, *L. variabilis,* and *Collembolispora disimilis* which are currently transferred from *Cadophora* form a subclade with *C. yulongensis* and two species of *Collembolispora*; *Cadophora fascicularis* clusters with species of *Mycochaetophora* in a distinct lineage. Thus, the currently circumscribed genus could be split into separate genera, but the introduction of more satisfying generic concepts depends on more phylogenetically related taxa in *Ploettnerulaceae* being involved.

Although *Cadophora* species are often encountered in cold environments, especially in the polar regions, most of them are psychrotolerant and have an optimum growth temperature (OGT) near or above 20 °C (Blanchette et al. 2021). The only psychrophilic species reported is *C. antarctica* which was isolated from a soil sample in King George Island (Antarctica) and had an OGT of 15 °C (Crous et al. 2017). Travadon et al. (2015) hypothesized that the geographic distribution patterns of *Cadophora* species in North America might reflect their adaptation to the contrasting environments: species recovered from cooler areas normally had an OGT at 20 °C and ones isolated from warmer regions tended to grow well at 25 °C. In our study, strains isolated from samples of Dagu Glacier (DG5, DG21, DG1048, DG1073, DG1087, DG1105 and DG1156), Mingyong Glacier (MY527, MY588, MY589, MY873) and Yulong Glacier (YL73) all had optimum growth rates at 20 °C, while others isolated from the same sampling sites had an OGT at 25 °C. Besides, strains being identified as the same species (*C. qinghai-tibetana*) have different OGTs (ranging from 20 °C to 25 °C). Environmental adaptations of fungal strains might be affected by many factors, such as temperature, humidity, radiation, and substrates. They have to evolve complex abilities to survive in adverse environments. Therefore, it is necessary to test more physiological, biochemical characteristics or perform genome analyses to illustrate adaptation mechanisms of this important fungal group.

## Conclusions

Our study shows a very high diversity of *Cadophora* in the marine glaciers of Qinghai-Tibet Plateau and we described seven *Cadophora* species new to science. With more species involved, the genus has become apparently paraphyletic and requires phylogenetic reconstruction. Thus, more comprehensive sampling is necessary for the creation of new generic concepts which could accommodate species which deviate morphologically and phylogenetically in this important fungal group.

## Data Availability

All sequence data generated for this study (Table 2) can be accessed via GenBank: https://www.ncbi.nlm.nih.gov/genbank/. Alignments are available at TreeBase (http://www.treebase.org) and available online at https://doi.org/10.6084/m9.figshare.20230977.v1

## Abbreviations

BI: Bayesian inference
BP: Bootstrap
CI: Consistency index
CGMCC: China General Microbiological Culture Collection Center
DIC: Differential interference contrast
HBU: Mycological Herbarium of Hebei University
HI: Homoplasy index
ITS: The internal transcribed spacer
LSU: The large ribosomal subunit (28S)
MCMC: Markov Chain Monte Carlo sampling
MEA: Malt extract agar
ML: Maximum likelihood
MP: Maximum Parsimony
NCBI: National Center for Biotechnology Information
OA: Oatmeal agar
PDA: Potato dextrose agar
PP: Posterior probabilities
RC: Rescaled consistency index
RI: Retention index
s.lat.: Sensu lato
s.str.: Sensu stricto
TEF: Translation elongation factor 1-α
TL: Tree length
TUB: β-Tubulin
UP: Unweighted parsimony

## References

[CR1] Alves A, Crous PW, Correia A, Phillips AL (2008). Morphological and molecular data reveal cryptic speciation in *Lasiodiplodia theobromae*. Fungal Divers.

[CR2] Bien S, Damm U (2020). *Arboricolonus simplex* gen. et sp. nov. and novelties in *Cadophora*, *Minutiella* and *Proliferodiscus* from *Prunus* wood in Germany. MycoKeys.

[CR3] Blanchette RA, Held BW, Jurgens JA, McNew DL, Harrington TC, Duncan SM, Farrell RL (2004). Wood-destroying soft rot fungi in the historic expedition huts of Antarctica. Appl Environ Microbiol.

[CR4] Blanchette RA, Held BW, Arenz BE, Jurgens JA, Baltes NJ, Duncan SM, Farrell RL (2010). An Antarctic hot spot for fungi at Shackleton’s historic hut on Cape Royds. Microb Ecol.

[CR5] Blanchette RA, Held BW, Hellmann L, Millman L, Büntgen U (2016). Arctic driftwood reveals unexpectedly rich fungal diversity. Fungal Ecol.

[CR6] Blanchette RA, Held BW, Jurgens J, Stear A, Dupont C (2021). Fungi attacking historic wood of Fort Conger and the Peary Huts in the High Arctic. PLoS ONE.

[CR7] Chen X, Cui P, Yang Z, Qi YQ (2005). Change in glaciers and glacier lakes in Boiqu river basin, middle Himalayas during last 15 years. J Glaciol Geocryol.

[CR8] Cheng GD (1998). Glaciology and geocryology of China in the past 40 years: progress and prospect. J Glaciol Geocryol.

[CR9] Conant NF (1937). The occurrence of a human pathogenic fungus as a saprophyte in nature. Mycologia.

[CR10] Crous PW, Wingfield MJ, Burgess TI, Carnegie AJ, Hardy GSJ, Smith D, Groenewald JZ (2017). Fungal Planet description sheets: 625–715. Persoonia Mol Phylog Evol Fungi.

[CR11] Crous PW, Wingfield MJ, Schumacher RK, Akulov A, Bulgakov TS, Carnegie AJ, Groenewald JZ (2020). New and Interesting Fungi. 3. Fungal Syst Evol.

[CR12] Day MJ, Currah RS (2011). Role of selected dark septate endophyte species and other hyphomycetes as saprobes on moss gametophytes. Botany.

[CR13] Day MJ, Hall JC, Currah RS (2012). Phialide arrangement and character evolution in the helotialean anamorph genera *Cadophora* and *Phialocephala*. Mycologia.

[CR14] Di MS, Calzarano F, Osti F, Mazzullo A (2004). Pathogenicity of fungi associated with a decay of kiwifruit. Australas Plant Pathol.

[CR15] Durán P, Barra PJ, Jorquera MA, Viscardi S, Fernandez C, Paz C, Bol R (2019). Occurrence of soil fungi in Antarctic pristine environments. Front Bioeng Biotechnol.

[CR16] Edler D, Klein J, Antonelli A, Silvestro D (2019). raxmlGUI 2.0 beta: a graphical interface and toolkit for phylogenetic analyses using RAxML. Methods Ecol Evol.

[CR17] Egidi E, Delgado-Baquerizo M, Plett JM, Wang J, Eldridge DJ, Bardgett RD, Singh BK (2019). A few Ascomycota taxa dominate soil fungal communities worldwide. Nat Commun.

[CR18] Ekanayaka AH, Hyde KD, Gentekaki E, McKenzie EHC, Zhao Q, Bulgakov TS, Camporesi E (2019). Preliminary classification of *Leotiomycetes*. Mycosphere.

[CR19] Furbino LE, Godinho VM, Santiago IF, Pellizari FM, Alves T, Zani CL, Rosa LH (2014). Diversity patterns, ecology and biological activities of fungal communities associated with the endemic macroalgae across the Antarctic Peninsula. Microb Ecol.

[CR20] Gams W (2000). *Phialophora* and some similar morphologically little–differentiated anamorphs of divergent ascomycetes. Stud Mycol.

[CR21] Gonçalves VN, Vaz AM, Rosa CA, Rosa LH (2012). Diversity and distribution of fungal communities in lakes of Antarctica. FEMS Microbiol Ecol.

[CR22] Gramaje D, Mostert L, Armengol J (2011). Characterization of *Cadophora luteo-olivacea* and *C. melinii* isolates obtained from grapevines and environmental samples from grapevine nurseries in Spain. Phytopathol Mediterr.

[CR23] Harrington TC, McNew DL (2003). Phylogenetic analysis places the *Phialophora*-like anamorph genus *Cadophora* in the *Helotiales*. Mycotaxon.

[CR24] Hyde KD, Dong Y, Phookamsak R, Jeewon R, Bhat DJ, Jones EB, Sheng J (2020). Fungal diversity notes 1151–1276: taxonomic and phylogenetic contributions on genera and species of fungal taxa. Fungal Diversity.

[CR25] Johnston PR, Quijada L, Smith CA, Baral HO, Hosoya T, Baschien C, Townsend JP (2019). A multigene phylogeny toward a new phylogenetic classification of *Leotiomycetes*. IMA Fungus.

[CR26] Jurgens JA, Blanchette RA, Filley TR (2009). Fungal diversity and deterioration in mummified woods from the ad Astra Ice Cap region in the Canadian High Arctic. Polar Biol.

[CR27] Katoh K, Standley DM (2013). MAFFT Multiple Sequence Alignment Software Version 7: improvements in performance and usability. Mol Biol Evol.

[CR28] Kumar S, Stecher G, Tamura K (2016). MEGA7: molecular evolutionary genetics analysis version 7 for bigger datasets. Mol Biol Evol.

[CR29] Lagerberg T, Lundberg G, Melin E (1927). Biological and practical researches into blueing in pine and spruce. Sven Skogsvardsforen Tidskr.

[CR30] Lim HJ, Nguyen TTT, Lee HB (2021). Six newly recorded fungal taxa from freshwater niche in Korea. Mycobiology.

[CR31] Maciá-Vicente JG, Piepenbring M, Koukol O (2020). Brassicaceous roots as an unexpected diversity hot-spot of helotialean endophytes. IMA Fungus.

[CR32] Marvanová L, Pascoal C, Cássio F (2003). New and rare hyphomycetes from streams of Northwest Portugal. Part I. Cryptogam Mycol.

[CR33] Nagano Y, Miura T, Nishi S, Lima AO, Nakayama C, Pellizari VH, Fujikura K (2017). Fungal diversity in deep-sea sediments associated with asphalt seeps at the Sao Paulo Plateau. Deep Sea Res Part II.

[CR34] Navarrete F, Abreo E, Martínez S, Bettucci L, Lupo S (2011). Pathogenicity and molecular detection of Uruguayan isolates of *Greeneria uvicola* and *Cadophora luteo-olivacea* associated with grapevine trunk diseases. Phytopathol Mediterr.

[CR35] Nisikado Y, Matsumoto H, Yamuti K (1934). Studies on a new *Cephalosporium*, which causes the stripe disease of wheat. Bericht des Ohara Instituts fur Landwirtschaftliche Forschungen.

[CR36] Rayner RW (1970). A mycological colour chart.

[CR37] Ronquist F, Huelsenbeck JP (2003). MrBayes 3: Bayesian phylogenetic inference under mixed models. Bioinformatics.

[CR38] Shi YF, Xie ZC (1964). The basic characteristics of modern glaciers in China. Acta Geogr Sin.

[CR39] Shi YF, Huang MH, Yao TD (2000). Glacier and environment in China—now, past and future.

[CR40] Su YY, Qi YL, Cai L (2012). Induction of sporulation in plant pathogenic fungi. Mycology.

[CR41] Sugar D, Spotts RA (1992). Sources of inoculum of *Phialophora malorum*, causal agent of side rot of pear. Phytopathology.

[CR42] Swofford DL (2002). PAUP*. Phylogenetic analysis using parsimony (and other methods). Version 4.

[CR43] Travadon R, Lawrence DP, Rooney-Latham S, Gubler WD, Wilcox WF, Rolshausen PE, Baumgartner K (2015). *Cadophora* species associated with wood-decay of grapevine in North America. Fungal Biol.

[CR44] Vilgalys R, Hester M (1990). Rapid genetic identification and mapping of enzymatically amplified ribosomal DNA from several *Cryptococcus* species. J Bacteriol.

[CR45] Walsh E, Duan W, Mehdi M, Naphri K, Khiste S, Scalera A, Zhang N (2018). *Cadophora meredithiae* and *C. interclivum*, new species from roots of sedge and spruce in a western Canada subalpine forest. Mycologia.

[CR46] Wang L, Zhuang WY (2004). Designing primer sets for amplification of partial calmodulin genes from *Penicillia*. Mycosystema.

[CR47] Wang M, Jiang X, Wu W, Hao Y, Su Y, Cai L, Liu X (2015). Psychrophilic fungi from the world's roof. Persoonia Mol Phylog Evol Fungi.

[CR48] White T, Bruns T, Lee S, Taylor J (1990). Amplification and direct sequencing of fungal ribosomal RNA genes for phylogenetics. PCR Protocols Guide Methods Appl.

[CR49] Xu LL, Li F, Xie HY, Liu XZ (2009). A novel method for promoting conidial production by a nematophagous fungus, *Pochonia chlamydosporia* AS6.8. World J Microbiol Biotechnol.

[CR50] Yao TD, Liu SY, Pu JC (2004). Retreat of high asia glacier and its affection to water resources in Northeast of China. Sci China (ser d).

[CR51] Zhang T, Zhao L, Yu C, Wei T, Yu L (2017). Diversity and bioactivity of cultured aquatic fungi from the High Arctic region. Adv Polar Sci.

